# Translation and Adaptation of the Child and Youth Resilience Measure-Revised and Rugged Resilience Measure: A Mixed-Method Study Among Adolescents in Nepal

**DOI:** 10.1007/s10578-025-01944-x

**Published:** 2025-12-05

**Authors:** Rakesh Singh, Kia-Chong Chua, Sagun Ballav Pant, Rajesh Paudel, Kamal Gautam, Nagendra Prasad Luitel, Emily Garman, Georgia Eleftheriou, Syed Shabab Wahid, Brandon A. Kohrt, Philip Jefferies, Mark J.D. Jordans, Crick Lund

**Affiliations:** 1 Research Department, Transcultural Psychosocial Organization Nepal, Baluwatar, Kathmandu, Nepal; 2 Centre for Global Mental Health, Health Service & Population Research Department, Institute of Psychiatry, Psychology and Neuroscience, King’s College London, London, UK; 3 Biostatistics & Health Informatics, Institute of Psychiatry, Psychology and Neuroscience, King’s College London, London, UK; 4 Department of Psychiatry & Mental Health, Institute of Medicine, Tribhuvan University, Kathmandu, Nepal; 5 Program Department, Transcultural Psychosocial Organization Nepal, Baluwatar, Kathmandu, Nepal; 6 Center for Global Mental Health Equity, Department of Psychiatry and Behavioural Health, The George Washington University, Washington, D.C, USA; 7 Department of Psychiatry and Mental Health, Alan J Flisher Centre for Public Mental Health, University of Cape Town, Cape Town, South Africa; 8 Department of Global Health, School of Health, Georgetown University, Washington, D.C, USA; 9 Mayo Campus, Atlantic Technological University, Castlebar, Ireland; 10 Resilience Research Centre, Dalhousie University, Halifax, Canada

**Keywords:** Resilience, Psychological, Adaptation, Adolescents, Nepal

## Abstract

Resilience, the capacity to adapt positively in adversity, is a key protective factor for adolescent well-being, particularly for depression and anxiety, which are highly prevalent among adolescents in Nepal. Accurate measurement across cultural contexts is essential to identify at-risk adolescents and understand protective mechanisms. This study culturally adapted and evaluated the psychometric properties of the Child and Youth Resilience Measure–Revised (CYRM-R) and Rugged Resilience Measure (RRM) in Nepal to ensure cultural relevance, reliability, and validity. This mixed-method study focused on poverty-affected adolescents in Kathmandu, using focus group discussions, cognitive interviews, pilot assessments, and a cross-sectional survey. The findings indicated Nepali versions of CYRM-R and RRM were acceptable, comprehensible, and relevant based on qualitative feedback. Most items showed item-total correlations between 0.2 and 0.5, indicating good discrimination, and internal consistency was satisfactory (α and ω > 0.7). Exploratory and confirmatory factor analyses supported a unidimensional structure, with an alternative two-factor solution explored for CYRM-R. Test-retest reliability was moderate overall, with some subscales less consistent. Both tools demonstrated strong psychometric properties, including face, content, convergent, and known-groups validity. The Nepali CYRM-R and RRM provide culturally robust tools for assessing adolescent resilience, supporting researchers, educators, and policymakers in designing targeted interventions.

## Introduction

Resilience, defined as the capacity to navigate, utilize, and negotiate access to resources that sustain well-being within one’s social and cultural ecology, has emerged as a critical factor in understanding positive adaptation among adolescents exposed to adversity [11–13]. Adolescents with higher resilience demonstrate greater ability to cope with challenges are less likely to develop depression or anxiety [10, 14–18], and are more likely to develop into resilient adults equipped with skills to navigate aversity [19]. Supporting adolescent resilience is therefore is vital for fostering their overall well-being and developmental success.

A systematic review focused on resilience and mental health in children and adolescents revealed differences in resilience outcomes across various socio-cultural settings and highlighted distinct processes specific to each context that contribute to these outcomes, particularly in low- and middle-income countries [20]. Singh and colleagues [21] reported that a significantly low proportion (15.4%) of Nepalese adolescents reported high resilience. The study used a standardized measure of resilience which had good psychometric properties in Nepalese adolescents [22]; however, the measure included 88 items, making it lengthy and difficult to administer, particularly among younger adolescents or those with lower comprehension.

Depression and anxiety are leading contributors to the global disease burden among young people [1] and are highly prevalent among Nepalese adolescents [2–5]. Nepali adolescents face multiple stressors, including poverty, political instability, natural disasters, social restrictions, domestic violence, and limited access to mental health resources [4–6]. Resilience, the capacity to overcome challenges, has been highlighted as a key protective factor, with higher resilience associated with lower risk of depression and anxiety [7–10].

Globally, resilience is measured using various tools depending on conceptualization, with modern approaches adopting multi-systemic perspectives that consider both internal and external protective factors [23–24]. Existing measures often focus on either internal strengths or external resources, limiting the ability to capture resilience holistically [25]. The Child and Youth Resilience Measure-Revised (CYRM-R) tool and Rugged Resilience Measure (RRM) are currently among the most suitable tools for assessing resilience because, together, they address limitations noted in existing resilience measures that focus narrowly on internal traits or coping characteristics. The CYRM-R was designed to reflect contemporary multisystemic models of resilience by capturing the external social, cultural, and relational resources that support adaptive functioning in the face of adversity, and was validated using Rasch analysis to ensure robust measurement of these contextual assets [26]. In contrast, the RRM was developed in response to critiques that external-resource measures alone do not fully account for individual capacities, and also in response to a lack of a brief and coherent measure of these internal “rugged” personal qualities that facilitate positive adaptation [27]. The CYRM-R and the RRM demonstrate strong psychometric properties, including high internal consistency, reliability, and evidence of construct validity, supporting their use in adolescent populations [26–27]. Used together, these measures offer complementary coverage of both internal characteristics and external supports, providing a more comprehensive, theoretically aligned, and multidimensional assessment of resilience than other available instruments, capturing intra-individual, interpersonal, and societal influences, which is especially relevant for adolescents in poverty-affected contexts.

While both measures have been used and validated across diverse cultural settings, they have not yet been adapted for Nepal. Nepal’s collectivist culture emphasizes family, community, and religious values, which may influence adolescents’ perceptions of support and agency. Spiritual practices and religious rituals often serve as coping mechanisms, fostering hope and emotional stability [28]. Socio-economic disparities, recurring natural disasters such as earthquakes and floods, and intergenerational trauma [29] may also shape resilience differently than in the original contexts of these instruments.

Given the lack of brief, culturally appropriate resilience measures in Nepal, particularly for adolescents exposed to poverty, this study adapted the CYRM-R and RRM for Nepalese adolescents living in poverty-affected areas of the Kathmandu Valley. The study evaluates their psychometric properties, including reliability and validity, to provide tools capable of capturing multi-dimensional resilience that can inform research, interventions, and policy in this context.

## Methods

### Participants and Procedure

This mixed-method study was embedded within a larger project *Improving Adolescent Mental Health by Reducing the Impact of Poverty (ALIVE)* aiming to develop and evaluate an intervention that addresses multidimensional poverty and self-regulation to prevent depression and anxiety among adolescents living in urban poverty [30]. One of the objectives of the ALIVE project included an adaptation of the tools to measure outcomes, mediators, and implementation parameters of the intervention. This study as a part of the ALIVE-instrument adaptation study was conducted among adolescents ages 11–17 years enrolled in public secondary schools of Budhanilkantha municipality and Kathmandu metropolitan city in Kathmandu Valley between August-October 2023.

Budhanilkantha municipality in Kathmandu Valley is densely populated, with adolescents affected by poverty, as shown by the ALIVE formative findings. The ALIVE intervention selected adolescents from public secondary schools (PSS) in Budhanilkantha and its adjacent wards (six and seven). Of the 21 PSS in the study area, 10 were excluded due to small student populations (grades 6–8 < 100) or proximity (< 1 km). These excluded schools were purposively chosen for the ALIVE-instrument adaptation study.

Focus group discussions (FGDs) and cognitive interviews were conducted with 112 adolescents in four PSS, with all sessions audio-recorded. A pilot assessment followed, using adapted Nepali tools in face-to-face interviews with 67 adolescents (ages 11–17) via the Open Data Kit (ODK) platform. Of these, 62 were identified as living in poverty using an 8-item poverty screening tool adapted from the multi-dimensional poverty index. These adolescents completed the full questionnaire for psychometric analysis. A cross-sectional survey was later conducted (February-March 2024) with 635 adolescents from eight PSS, screening 491 as living in poverty; with one dropped out, 490 completed full face-to-face interviews via ODK. The inclusion criteria for the participants in the survey included adolescents aged 13–15 years, enrolled in grades 6–8 in public secondary schools, who screened positive on the poverty screening tool, and provided assent (and consent by their caregivers) for their participation in the study. Those with severe visual, hearing, or other marked impairments that prevent comprehension of the survey tools were excluded from the study. Data collection was led by trained research assistants, who received training from the lead author on research methods, ethics, consent, adverse event reporting, interviewing, and ODK data collection and storage. We used the STROBE cross-sectional reporting guidelines (see Appendix Table S1) [31]. The data collection method employed a researcher-administered face-to-face interview (reading items aloud and showing the printed glass scale for categories of responses for the measures, including resilience and depression, and anxiety).

### Sample

Within the qualitative part of this study, a total of 11 FGDs (*n* = 68 adolescents) and nine cognitive interviews were conducted, involving adolescents aged 11–17. We selected 62 adolescents purposively in the pilot assessment from three schools, ensuring proportional distribution across schools, gender, and age groups (young adolescents aged 11–13 and old adolescents aged 14–17), with the mean age of the participants being 13.35 years (SD 1.29). Similarly, in the survey we purposively selected 635 adolescents from eight schools for poverty screening, ensuring proportional distribution across schools, gender, age (13–15 years), and grades (6–8), with at least 50 participants per school. The survey involved 490 adolescents (mean age 13.85 years, SD 0.73), 52.7% of whom were girls, for the analysis (see Appendix Table S2).

### Instruments

#### CYRM-R and RRM

The CYRM-R [27] is a 17-item, simpler measure of resilience from a socio-ecological framework, in children and adolescents in diverse cultural contexts, consisting of two subscales, including intra/interpersonal and caregiver/relational resilience. The CYRM-R for youths’ version was used in the study which is appropriate for ages ranging from 10 to 23 years. The responses on a five-point scale (Not at all – A lot) were coded such that higher scores indicated higher levels of resilience [33]. The RRM is a 10-item brief yet reliable tool for measuring psychological or internal resilience, demonstrating effectiveness across various global contexts [26]. Ten items constitute 10 internal qualities (e.g., self-belief, perseverance, optimism, etc.). It can evaluate internal protective factors alone or alongside social-ecological factors for a comprehensive view of resilience and overall quality of life. Similar to CYRM-R, the items of RRM are measured on a five-point scale with coding such that higher scores are interpreted as having higher resilience.

The CYRM-R demonstrates strong psychometric properties. The original validation reported good internal consistency (Cronbach’s α = 0.82) and robust Rasch-model fit, indicating unidimensionality, satisfactory fit statistics, strong internal reliability (person–separation index), and no evidence of item bias or problematic local dependency [26]. Subsequent use of the English version has further supported its reliability, with a Cronbach’s alpha of 0.88 reported in an adolescent sample [58]. The RRM similarly shows strong reliability and validity evidence. Initial validation reported Cronbach’s α = 0.87 and McDonald’s ωh = 0.83, alongside clear support for content validity through expert review, and evidence of construct, concurrent, convergent, and discriminant validity [27]. The measure also demonstrated incremental validity, adding unique explanatory power beyond the CYRM-R in hierarchical analyses. Together, findings indicate that both instruments provide a reliable and valid assessment of resilience in adolescents and youths, ensuring their usability in both research and practice [32].

#### Other Measures

Depressive symptoms were assessed by the Patient Health Questionnaire Adolescent (PHQ-A) score and anxiety symptoms were assessed by the Generalized Anxiety Disorder-7 (GAD-7) score. The poverty score was assessed by the self-developed poverty screening tool. The Multi-Dimensional Poverty Index was adapted into an eight-item screening tool for the ALIVE study to identify adolescents experiencing multidimensional poverty in the local context [30]. The tool assessed household factors including income, dependency ratio, education levels of household members, access to private sanitation and kitchen facilities, and ownership of key assets. Items were weighted to calculate a total deprivation score, with scores ≥ 0.33 indicating poverty. Although formal psychometric testing has not been conducted, the tool was developed through expert consultation and piloted for comprehension and contextual relevance. Preliminary validation in Nepal showed that 62 of 67 pretest participants screened positive for poverty, with qualitative assessments supporting its construct and contextual relevance. The validated tools in the context of Nepali adolescents, including the PHQ-A and the GAD-7 [45], as part of the Measure of Mental Health among Adolescents and Young People at the Population Level, were used to assess depressive and anxiety symptom scores, respectively, with higher scores indicating greater symptom severity (both Cronbach’s α = 0.79).

#### Adaptation of Instruments

The CYRM-R and RRM were translated into Nepali following established guidelines for cross-cultural translation [34]. Initially, two bilingual experts—a psychiatrist and a psychologist—translated the items from English to Nepali. An independent bilingual expert then back-translated the Nepali versions into English. The lead author of the original CYRM-R and RRM reviewed the back translations to ensure conceptual equivalence and accurate representation of the intended constructs.

The translated items were evaluated for comprehensibility, relevance, acceptability, response format, and completeness. Subsequent review by a psychiatrist and two mental health researchers led to structural modifications, including rephrasing statements as questions, simplifying language, adding examples, and incorporating visual (glass) scales as response categories.

To further assess clarity and appropriateness, feedback was obtained from adolescents through focus group discussions and cognitive interviews, considering variations in age, gender, and ethnicity. Items were revised based on this feedback to enhance contextual relevance and ensure that the translated tools were clear, culturally appropriate, and understandable for the target population.

Although content validity was not assessed quantitatively prior to data collection, a retrospective evaluation was conducted using a panel of 10 experts in adolescent mental health and resilience. Ten subject-matter experts were asked retrospectively to evaluate each item on two dimensions. ‘Essentiality’ was rated using three response options: “Essential” (score = 2), “Useful but not essential” (score = 1), and “Not necessary” (score = 0). ‘Relevance’ was rated on a four-point scale: “Highly relevant” (3), “Quite relevant” (2), “Somewhat relevant” (1), and “Not relevant” (0). For essentiality, the Content Validity Ratio (CVR) for each item was computed using Lawshe’s formula. For relevance, the Item-level Content Validity Index (I-CVI) was calculated as the proportion of experts rating an item as “Quite relevant” or “Highly relevant” (scores 2 or 3). The Scale-level CVI (S-CVI/Ave) was derived as the mean of all I-CVI values across the scale items. Because the expert ratings were obtained after instrument administration (a post-hoc step), we explicitly treated this exercise as a retrospective content validation and report it accordingly.

Overall, the post-hoc content validation indicated strong agreement among experts for both scales. CVR values across items ranged from 0.60 to 1.00 with a mean CVR of 0.87, indicating that most items were judged essential by a substantial majority of experts. I-CVI values ranged from 0.80 to 1.00 for items of CYRM-R and RRM, and the scale-level S-CVI (average of item I-CVIs) was 0.95 and 0.98 for CYRM-R and RRM, respectively, exceeding thresholds for excellent content validity. Two items—CYRM item 2 and RRM item 10—had CVR values marginally below the Lawshe critical value for *N* = 10 (critical CVR ≈ 0.62); despite this, both items demonstrated acceptable I-CVI values (≥ 0.80), indicating that experts still judged these items to be relevant even if slightly fewer rated them as strictly “essential.” The post-hoc assessment supported the appropriateness and relevance of the items for measuring resilience-related constructs in Nepali adolescents.

### Data Analysis

The qualitative findings from FGDs and cognitive interviews conducted as per van Ommeren’s guidelines informed the cultural translation and adaptation of the CYRM-R and RRM tools. The psychometric properties of the adapted versions, including descriptive statistics such as mean, standard deviation (SD), skewness, and kurtosis, were assessed using data from the pilot assessment (*N* = 62). Descriptive statistics for both tools, including mean, standard deviation (SD), skewness, kurtosis, and item-total correlation (ITC), were calculated. The internal consistency of the scales and sub-scales was measured using mean inter-item correlations and Cronbach’s alpha, following the original factor structure of the tools. For the larger cross-sectional sample (*N* = 472 for CYRM-R, *N* = 486 for RRM), the psychometrics, including polychoric correlations, polychoric item-total correlation (ITC), polychoric mean-inter item correlations, Cronbach’s alpha, McDonald’s omega, exploratory factor analysis (EFA), and confirmatory factor analysis (CFA), and invariance measurements across gender were conducted to examine the factor structure of the Nepali versions. Internal consistency of the CYRM-R was first assessed for the total scale using Cronbach’s α and McDonald’s ω, showing that the 17 items formed a reliable set. Factor analysis (EFA followed by CFA) was then conducted to examine the latent structure in this sample. Internal consistency for each factor/subscale was calculated only after factor loadings were identified, ensuring that reliability estimates corresponded to empirically derived factors. EFA sample size recommendations were followed [35–38]. The Kolmogorov-Smirnov test indicated a non-normal data distribution. To examine the factorial validity of the resilience measures, the total sample was randomly divided a priori into two halves (50/50). An Exploratory Factor Analysis (EFA) using Principal Axis Factoring with Direct Oblimin rotation was conducted on the first half of the sample. The resulting structures were then validated through Confirmatory Factor Analysis (CFA). Before performing EFA, Kaiser Meyer-Olkin (KMO) and Bartlett’s Test of Sphericity were assessed to test the data’s suitability for factor exploration. We tested the measurement invariance of the 1-factor models for CYRM-R and RRM across gender (male and female) using multi-group CFAS. As for all CYRM-R and RRM items were rated on a 5-point Likert scale, sparse response categories by gender were collapsed to ensure sufficient observations for ordinal CFA for measurement invariance assessment. Measurement invariance across gender was examined using WLSMV CFA for ordinal data, including the 1-factor RRM, a 1-factor CYRM-R, and a 2-factor CYRM-R model. Invariance testing followed the standard sequence—configural, metric, and scalar (threshold)—assessed using ΔCFI < 0.01 and ΔRMSEA< 0.015. Multiple methods, such as the scree plot, Kaiser’s criterion, parallel analysis, and Velicer’s Minimum Average Partial (MAP) test, helped determine the number of factors to retain. Convergent validity was assessed using Spearman’s rank-order correlations (ρ) between resilience scores (CYRM-R, personal resilience [PR], relational resilience [RR], and RRM) and related constructs including depressive symptoms, assessed by the PHQ-A score, anxiety symptoms, assessed by the GAD-7 score, and poverty (poverty screening score), given the ordinal and non-normal data for the total sample. Known-groups validity was examined using the Wilcoxon rank-sum test (W) to compare resilience scores by gender, with accompanying effect size r. Additionally, the test-retest reliability was explored with a sub-sample of 20 adolescents from the pilot assessment, reassessed after 2 weeks. Intraclass correlation coefficients (ICC) were used to assess reliability, with confidence intervals indicating poor, moderate, good, or excellent reliability based on the ICC values [39]. While data cleaning, descriptive statistics, test-retest reliability, and generation of CFA diagrams were performed, the quantitative analysis was conducted using SPSS version 25 and AMOS v. 31. All other statistical analyses—including internal consistency assessment, EFA, CFA, measurement invariance testing across gender, and preliminary validity analyses—were conducted in R (version 4.5.1) using appropriate packages for ordinal data and robust WLSMV estimation methods.

### Ethics

The study, part of ALIVE-instrument adaptation and validation, received ethical approval from King’s College London (HR/DP-22/23-33982; RESCM-23/24-33982; HR/DP-23/24-38680; RESCM-23/24-38680) and Nepal Health Research Council (Ref 988; protocol reg. no. 487/2022 P; Ref 896; protocol reg. no. 661/2023). Data collection permission was granted by municipal education units and school principals. Adolescents and caregivers were informed about the study, and signed consent and assent forms were obtained before data collection.

## Results

Overall, the FGDs and CIs results showed that most items translated into Nepali for both tools were acceptable, easily understandable, relevant, easy to formulate responses to, and complete for various groups of adolescents. The detailed findings related to the adaptation of the items of CYRM-R and RRM have been described in Appendix Table S3 and Table S4.

### Descriptive Statistics of the Items of CYRM-R and RRM

The descriptive and item-level analyses suggest that most items on the CYRM-R and RRM scales performed adequately (see Table 1); they indicated generally high mean scores with substantial negative skewness, suggesting higher resilience among Nepali participants. Ordinal item–total correlations (ITCs) for the CYRM-R were moderate overall (0.20–0.40), indicating acceptable item discrimination, with the strongest associations observed for items assessing caregiver support, school belonging, and perceived safety.

Ordinal ITCs for the RRM items showed greater variability (–0.77 to 0.54). While several items demonstrated adequate correlations with the full scale (e.g., perseverance and problem-solving), others—particularly items related to emotional control, pride, and coping—showed weak or negative ITCs.

Internal consistency estimates for the CYRM-R and RRM scales demonstrated acceptable to strong reliability across both the EFA and CFA samples (Table 2). For the full 17-item CYRM-R, polychoric mean inter-item correlations (MIICs) were within the recommended range (0.28–0.32), with ordinal Cronbach’s α values of 0.87–0.89 and McDonald’s ω values of 0.84–0.91, indicating good overall internal consistency. As per the latent 2-factorial structure in the Nepali sample, the Personal Resilience subscale showed MIICs of 0.27–0.33, with α ranging from 0.78 to 0.83 and ω from 0.73 to 0.86, reflecting acceptable internal reliability across samples. The Relational Resilience sub-scale similarly demonstrated adequate internal consistency, with MIICs between 0.33 and 0.39, α values of 0.77–0.82, and ω values of 0.75–0.81. For the 10-item RRM scale, reliability indices were consistently strong. MIICs ranged from 0.34 to 0.39, while ordinal Cronbach’s α values were 0.84–0.86, and ω values were 0.81–0.86. Overall, these findings indicate satisfactory internal consistency for all scales and subscales, with largely comparable patterns across the EFA and CFA samples.

### Exploratory and Confirmatory Factor Analyses of the Nepali Version of CYRM-R and RRM

The 17 items of the CYRM-R and 10 items of RRM were analyzed using principal axis factoring (PAF) to assess data suitability for factor analysis. For the CYRM-R, the correlation matrix showed many coefficients above 0.3, with a Kaiser-Meyer-Olkin value of 0.82, exceeding the recommended 0.6. Bartlett’s Test of Sphericity was significant (*p* < 0.001), confirming the data’s suitability. For the RRM, the inspection of the correlation matrix revealed the presence of many coefficients of 0.3 and above and the Kaiser Meyer-Olkin value was 0.90 and Bartlett’s Test of Sphericity reached statistical significance (*p* < 0.001), supporting the factorability of the correlation matrix. The parallel analysis using principal axis factoring (PAF) on the polychoric correlation matrix showed four factors for CYRM-R and one factor for RRM. While the one-factor model for RRM was theoretically valid with support from parallel analysis, screeplot, the ratio of the first two eigenvalues (Kaiser’s criterion) [40–41], Velicer’s Minimum Average Partial (MAP), and theoretical construction. Therefore, the EFA was conducted to examine the dimensionality of the CYRM-R by exploring solutions with one to four factors and RRM by exploring the 1-factor solution. The EFA of the CYRM-R indicated that both one- and two-factor solutions were viable. In the one-factor model, factor loadings ranged from 0.35 to 0.71, with communalities between 0.12 and 0.51, supporting a general resilience factor (see Table 3). The two-factor solution accounted for conceptually meaningful subscales, with Factor 1 loadings ranging from 0.21 to 0.82 and Factor 2 loadings from 0.10 to 0.80; communalities ranged from 0.12 to 0.63. Several items exhibited moderate cross-loadings, but overall, the two factors were correlated at 0.59, indicating related but distinguishable dimensions of resilience. For the RRM, a unidimensional structure was supported, with loadings ranging from 0.43 to 0.77 and communalities from 0.18 to 0.59, consistent with a single resilience factor. While doing a comparison across the four models of CYRM-R (see Table S5–S7 in Appendix), the one-factor solution, which explained 33% of the variance, supported a reliable general factor (factor score correlation = 0.95) but did not provide subscale-level differentiation. The two-factor solution accounted for 38% of the variance, with most items loading clearly onto two conceptually interpretable factors representing personal and relational resilience, and factor score reliability ranging from 0.91 to 0.93, making it suitable for subscale-level analyses. The three-factor solution explained slightly more variance (43%) but included one factor with very few items and multiple cross-loadings, reducing interpretability. The four-factor solution explained 48% of the variance and achieved the best overall fit (RMSR = 0.04), yet high item complexity and low minimum factor score correlations (0.53–0.72) indicated unstable and difficult-to-interpret factors. Overall, while the two-factor solution provided the optimal balance between variance explained, interpretability, and factor score stability, the one-factor structure provided a general overall dimension of the external protective factor. Therefore, 1-factor and 2-factor structures for CYRM-R and a unidimensional structure for RRM were selected as the most appropriate structures for subsequent analyses in the Nepali sample, including CFA and invariance measurement across gender.

**Fig. 1:**
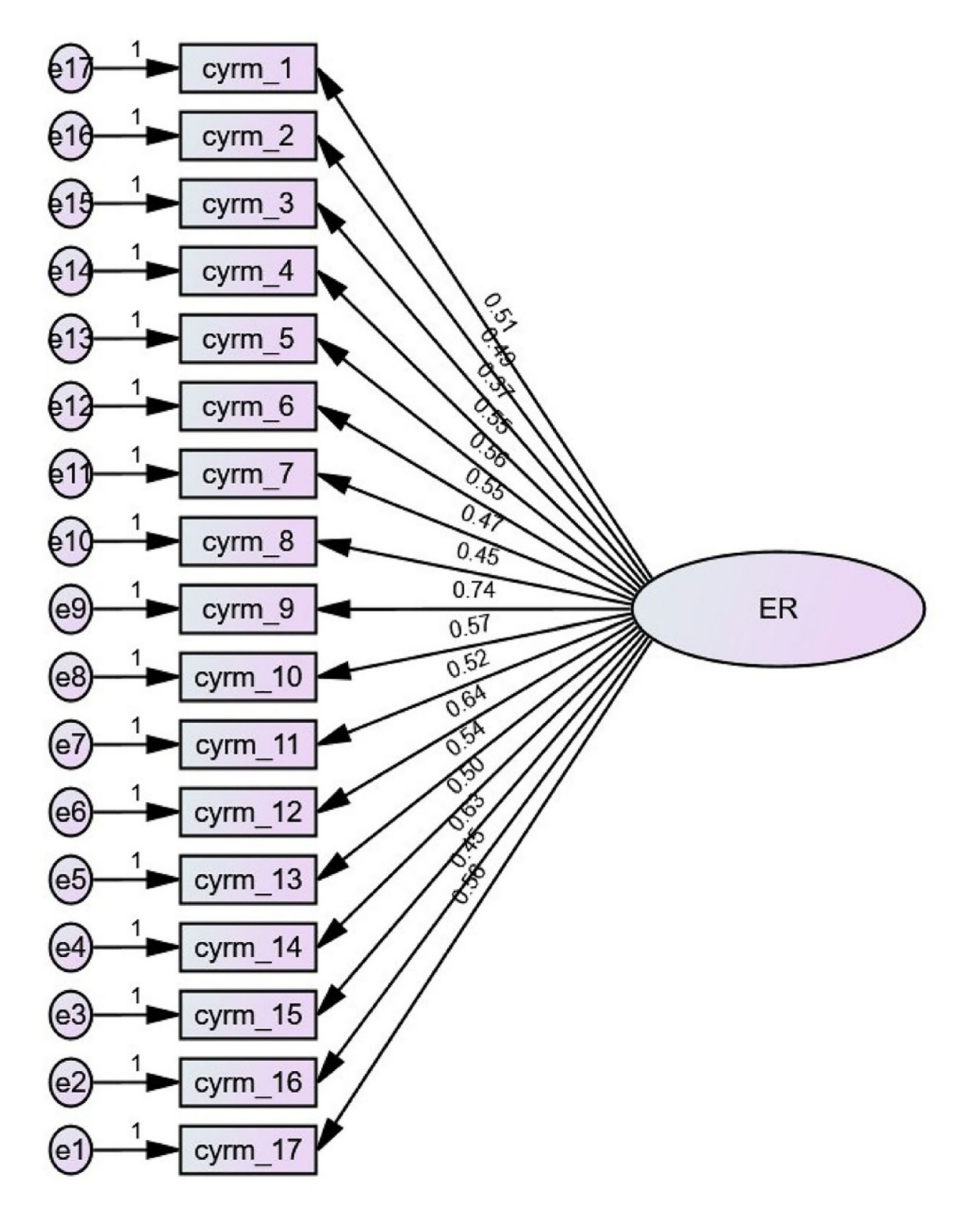
Path diagram showing one factor model of CYRMR. Note: e = error terms, ER = external resilience, cyrm denotes CYRM-R item numbers, factor loadings are standardized

**Fig. 2:**
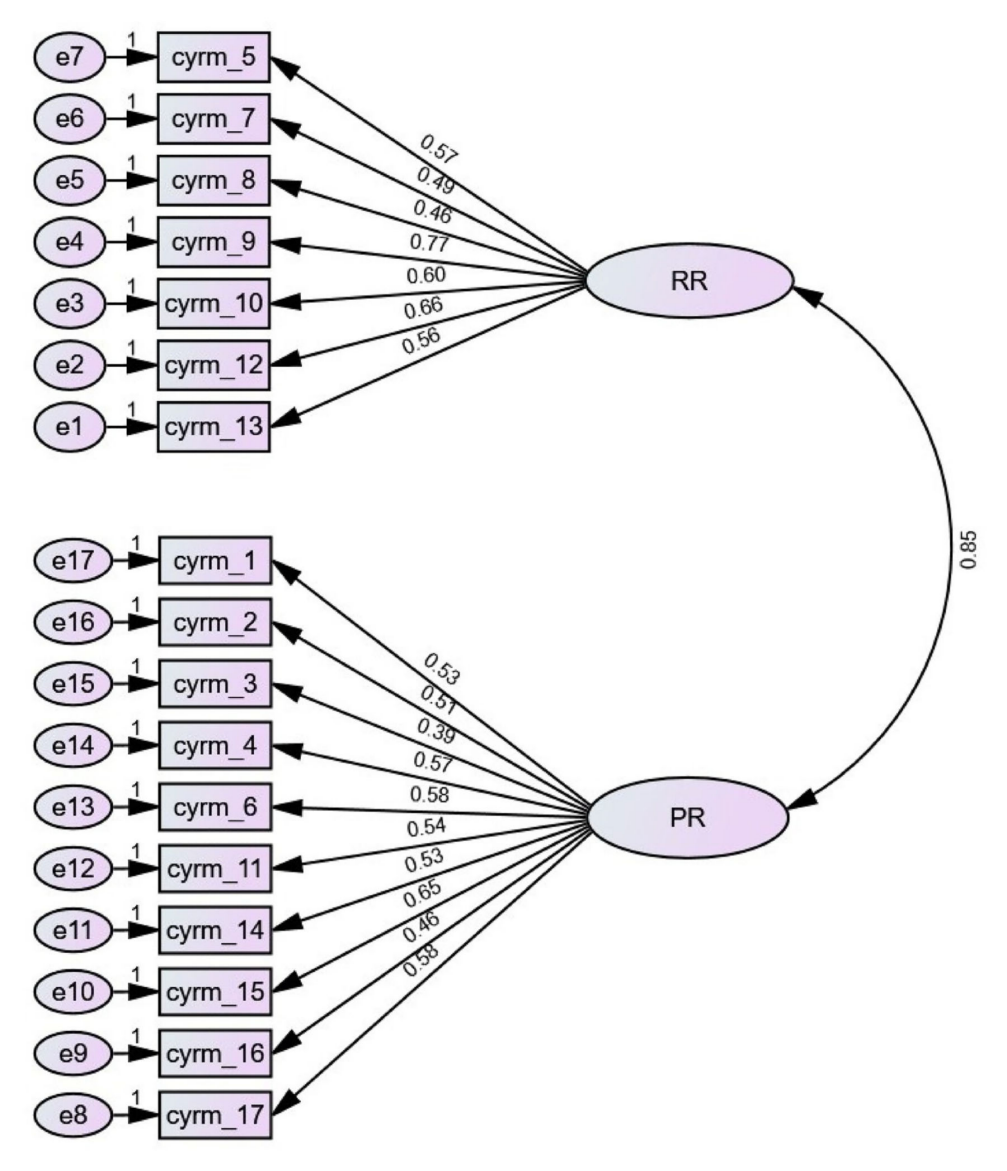
Path diagram showing two factor model of CYRM-R. Note: e = error terms, RR = relational resilience, PR = personal resilience, cyrm denotes CYRM-R item numbers, factor loadings are standardized

**Fig. 3:**
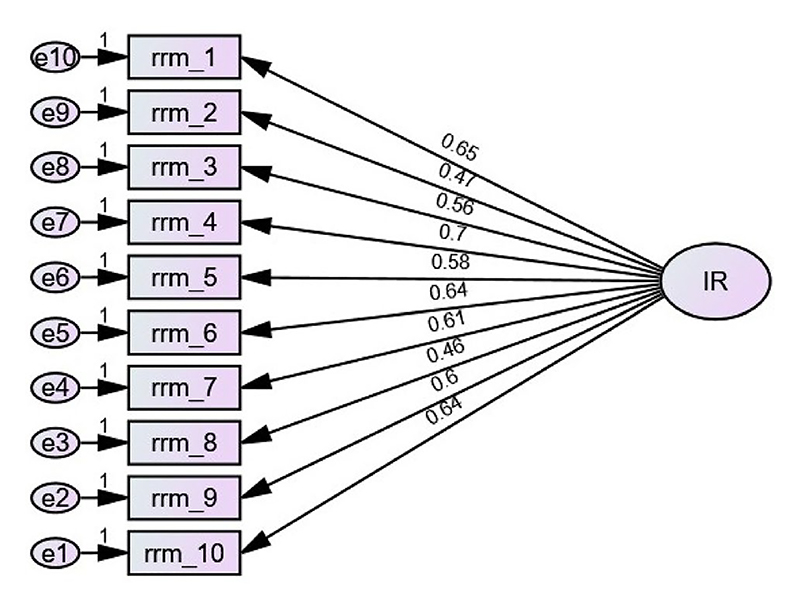
Path diagram showing one factor model of RRM. Note: e = error terms, IR = internal resilience, rrm denotes RRM item numbers, factor loadings are standardized

A CFA conducted using WLSMV estimation for ordinal data on the second half of the sample further validated the one-factor structure for RRM and both one-factor and two-factor structures for CYRM-R (Table 4). For the CYRM-R, the one-factor model showed marginal to acceptable fit (χ^2^/df = 2.26; CFI = 0.77; TLI = 0.74; RMSEA=0.10; SRMR = 0.08), while the two-factor model demonstrated slightly improved fit over the 1-factor model across all indices (χ^2^/df = 1.80; CFI = 0.79; TLI = 0.76; RMSEA=0.09; SRMR = 0.08), supporting the presence of two correlated subscales. The RRM one-factor model exhibited good to excellent fit (χ^2^/df = 1.11; CFI = 0.95; TLI = 0.93; RMSEA=0.06; SRMR = 0.05), indicating that a unidimensional structure adequately represents this scale. Overall, these results support both the one-factor parsimonious structure to assess general external resilience and two-factor latent structures for the CYRM-R to examine personal and relational resilience and a single-factor structure for the RRM in Nepali adolescents. The path diagrams for models of CYRM-R and RRM are shown in Figs. 1, 2 and 3.

### Invariance Measurement across Gender for CYRM-R Models

Measurement invariance across gender was examined for both the 1-factor and 2-factor CYRM-R models using ordinal CFA with WLSMV estimation. For the 1-factor model, the configural model demonstrated acceptable baseline fit (Df = 238; AIC = 361.62), indicating that the overall factor structure was similar across male and female participants. When constraining factor loadings in the metric model, the scaled chi-squared difference test showed no significant worsening of fit compared with the configural model (ΔDf = 16; ΔChi^2^ = 16.20; *p* = 0.439), with RMSEA=0.114. Fit index changes were minimal (ΔCFI = 0.008, ΔRMSEA=0.004), both within recommended cut-offs, supporting metric invariance. When item thresholds were further constrained to test scalar invariance, the difference test yielded ΔDf = 23; ΔChi^2^ = -15.72; *p* = 1.000, and fit index changes were ΔCFI = -0.011 and ΔRMSEA = -0.011. While the ΔCFI slightly exceeded the conventional 0.01 threshold, the ΔRMSEA remained within acceptable limits, suggesting that scalar invariance is marginally supported, and item thresholds can be considered largely equivalent across genders.

For the 2-factor model, the configural model also demonstrated acceptable baseline fit (Df = 236; AIC = 339.01), indicating equivalence of the two-factor structure across gender. Constraining factor loadings for the metric model resulted in ΔDf = 15; ΔChi^2^ = 16.998; *p* = 0.319, with RMSEA=0.122. The changes in fit indices were ΔCFI = 0.008 and ΔRMSEA = 0.005, supporting metric invariance. Constraining item thresholds for the scalar model yielded ΔDf = 23; ΔChi^2^ = -15.99; *p* = 1.000, with ΔCFI = -0.011 and ΔRMSEA = -0.012. Similar to the 1-factor model, these results indicate that scalar invariance is marginally supported, with minor deviations in thresholds across gender.

Overall, these findings indicate that for both the 1-factor and 2-factor CYRM-R models, factor structure and loadings were equivalent across male and female participants, while item thresholds show only minor differences, making comparisons of latent factor means across gender largely valid.

### Invariance Measurement across Gender for RRM Model

A multiple-group CFA was conducted to examine whether the 10-item RRM scale functioned equivalently across male (*n* = 118) and female (*n* = 125) participants. All items were treated as ordinal and analyzed using the WLSMV estimator with theta parameterization. Initial inspection of item distributions revealed sparse responses in extreme categories, particularly for items 1, 4, 5, 6, 8, 9, and 10. To address empty cells, response categories 1 and 2 were collapsed into a single lowest category, and categories 4 and 5 were collapsed into a single highest category, while maintaining the ordinal structure.

The configural model, in which all factor loadings were freely estimated across genders, demonstrated good fit: χ^2^(70) = 63.43, *p* = 0.697; RMSEA=0.000 (90% CI [0.000, 0.043]), p(RMSEA≤0.05) = 0.978; SRMR = 0.100; CFI = 1.000; TLI = 1.008. Standardized factor loadings were all statistically significant across groups, ranging from 0.323 to 0.815 in females and 0.414 to 0.755 in males, indicating that the latent structure of resilience was equivalent across genders, supporting configural invariance. Latent factor variances were comparable (female = 1.506, male = 1.644), suggesting similar variability in resilience. Threshold estimates, calculated after collapsing sparse categories, indicated minor differences across genders but were generally comparable, supporting the equivalence of the response scale.

Metric invariance was tested by constraining factor loadings to be equal across males and females. The model fit remained acceptable: χ^2^(79) = 86.02, *p* = 0.276; RMSEA=0.027 (90% CI [0.000, 0.059]), p(RMSEA≤ 0.05) = 0.857; SRMR = 0.111; CFI = 0.993; TLI = 0.993. All loadings remained significant and similar in magnitude to the configural model. Changes in fit indices relative to the configural model were minimal (ΔCFI = -0.004, ΔRMSEA = 0.000), both within conventional cut-offs (ΔCFI < 0.01, ΔRMSEA < 0.015), supporting metric invariance. This indicates that the relationship between the latent resilience factor and individual items is equivalent across male and female participants.

Scalar (threshold) invariance was assessed by additionally constraining item thresholds across groups. The model also demonstrated acceptable fit: χ^2^(88) = 98.35, *p* = 0.184; RMSEA=0.030 (90% CI [0.000, 0.061]), p(RMSEA≤ 0.05) = 0.811; SRMR = 0.114; CFI = 0.991; TLI = 0.992. Standardized factor loadings remained stable and significant, and thresholds were largely comparable across genders, indicating that participants with the same latent resilience level responded similarly to the items. The changes in fit indices relative to the metric model were small (ΔCFI = -0.004, ΔRMSEA = 0.003), supporting scalar invariance.

Overall, the 1-factor RRM model demonstrated full configural, metric, and scalar invariance across male and female adolescents. Factor loadings were substantial and positive (generally > 0.40), latent factor variances were comparable, and thresholds were largely equivalent. These findings indicate that the RRM scale measures resilience equivalently across genders, supporting the validity of comparing latent factor means and conducting latent variable analyses between male and female participants.

### Convergent and Known-Groups Validity

Spearman correlations were calculated to assess convergent validity among resilience scales and subscales (CYRM-RR, personal resilience [PR], relational resilience [RR], and RRM score), psychological distress (PHQA and GAD7 scores), and poverty (see Table 5). The resilience scales and subscales were moderately to strongly intercorrelated (CYRMR–PR: ρ = 0.899, CYRMR–RR: ρ = 0.898, PR–RR: ρ = 0.627; CYRMR–RRM: ρ = 0.676; PR–RRM: ρ = 0.655; RR–RRM: ρ = 0.567; all *p* < 0.001), supporting convergent validity. As expected, resilience measures were negatively correlated with psychological distress (PHQA ρ = -0.211 to -0.260; GAD7 ρ = -0.189 to -0.222; all *p* < 0.001) and poverty (ρ = -0.090 to -0.134; *p* < 0.05), indicating that higher resilience is associated with lower distress and socioeconomic disadvantage.

Known-groups validity was examined by comparing resilience scores across gender using Wilcoxon rank-sum tests. No significant gender differences were observed for CYRM-R (W = 30291, *p* = 0.817, *r* = 0.01), PR (W = 29446, *p* = 0.758, *r* = 0.01), RR (W = 31213, *p* = 0.411, *r* = 0.04) and RRM showed a non-significant trend (W = 32772, *p* = 0.069, *r* = 0.08), suggesting that the resilience scales function similarly across males and females.

Collectively, the findings provide evidence for the construct, convergent, and known-groups validity of the Nepali CYRM-R and RRM measures, while highlighting that the choice of factor structure (1-, or 2-factor for the CYRM-R should be guided by research objectives and interpretability.

### Test-Retest Reliability of the Nepali Version of CYRM-R and RRM

The findings related to the test-retest of the CYRM-R and RRM are presented in Table 6. For all resilience scales and sub-scales, the overall ICC indicated moderate reliability (ICC ranging from 0.61 to 0.73). When examined separately by gender, ICCs were generally higher in females than in males. For the CYRM-R total score, ICC was 0.48 (95% CI: -1.01, 0.87) in males and 0.76 (95% CI: -0.03, 0.94) in females. Personal resilience showed lower reliability in males (ICC = 0.27; 95% CI: -1.69, 0.82) compared with females (ICC = 0.74; 95% CI: -0.13, 0.94), whereas relational resilience was more stable in males (ICC = 0.81; 95% CI: 0.21, 0.95) than females (ICC = 0.68; 95% CI: -0.46, 0.92). For the RRM scale, ICC was 0.58 (95% CI: -0.28, 0.89) in males and 0.83 (95% CI: 0.35, 0.96) in females. These findings suggest that test–retest reliability is generally acceptable but variable across subscales and genders, and the small sample size (*n* = 10 per group) results in wide confidence intervals, indicating these results should be considered preliminary.

## Discussion

The overall findings related to the cultural translation and adaptation of the CYRM-R and RRM revealed that Nepali items of both resilience measures were acceptable, understandable, relevant, and complete to Nepalese adolescents. The use of pictures (glass scale) as response categories for the resilience measures in the Nepalese sample to maintain uniformity in the assessment was similar to that of the visual glass scale used in the study among Syrian refugees and Jordian host-community adolescents living in urban centers close to the Syrian border [42]. The use of visual scales is also supported by the suggestions made by developers of the tools to use pictorial scales in younger populations or those with comprehension challenges [33]. Nepalese adolescents reported that the water glass response scale helped them distinguish between various response options. Another major adaptation in the items of both CYRM-R and RRM in the Nepali version was in the format, i.e., statements were adapted into questions. These changes improved comprehension, as adolescents found it easier to understand and respond to items when framed as questions. The use of questions was congruent with the Hindi version of CYRM-R [43]. This was also in line with other studies identifying the need to minimize social bias linked with declarative sentences [44–45] and has been recognized as one of the important tool design elements in cultures with strong social hierarchies [46]. Other adaptations in the items of the tools included adding specific examples to clarify abstract concepts (e.g., defining “staying together in harmony” as helping, eating, and playing together; explaining “adapting to challenges” with scenarios like adjusting to a new school or reconciling with friends after a fight, etc.), adjusting wording for cultural and contextual relevance (e.g., explaining fairness in the community through behaviors like honesty and kindness; illustrating pride in achievements with relatable examples like winning in sports or cooking a good meal, etc.), and breaking down complex and key ideas into simpler, relatable terms to ensure better comprehension (e.g., defining feeling safe within the family with examples like not being scared or being able to socialize freely; explaining “coping with competing demands” by listing tasks such as tuition, homework, and cooking). These changes helped ensure Nepalese adolescents better understand and relate to the items more contextually. Post-hoc quantitative content validation demonstrated excellent expert agreement for both CYRM-R and RRM, with high CVR and I-CVI values indicating that the items are largely essential and relevant for assessing resilience-related constructs in Nepali adolescents.

The ITC for a majority of the items of the Nepali versions of CYRM-R and RRM indicated that these items within both tools discriminated well by the Nepalese sample, except for a few items of the RRM, such as those assessing pride and emotional control. This anomaly may be attributed to various socio-cultural norms that discourage open expression of emotions or emphasis on humility over individual achievement, and development variability in self-regulation during adolescence. This finding suggests that such items may capture a distinct facet of resilience that is less correlated with the overall scale in this context. Similarly, as the inter-item correlations were below 0.7 for total and subscales of the CYRM-R and RRM in the Nepalese sample, none of the items were removed from the Nepali version of the CYRM-R and RRM. The internal consistency of the different items of both CYRM-R and RRM among Nepalese adolescents was good. The CYRM-R has been used in diverse populations and has been an important measure of resilience as an external resource for adolescents in 14 countries [48]. The RRM, a relatively a new tool, was also found to be a reliable tool for measuring internal resilience in Indonesian populations [49–50] and South African youth populations [51].

The results of the exploratory and confirmatory factor analysis for the RRM in Nepalese adolescents showed a similar one-factor latent construct as per the original RRM tool [26]. A congruent, one-factor latent construct for RRM was observed in the Indonesian adult population [49]. While the RRM in Nepali adolescents demonstrated strong evidence for a unidimensional structure consistent with its original latent model, items 7 and 8 (“I am generally in control of my emotions”, “I take pride in things I have achieved”) showed a relatively lower loading in the Nepali sample. This may reflect cultural differences in how adolescents in Nepal perceive and express personal pride and emotions. In a collectivist cultural context that values modesty, interdependence, and social harmony, overt expressions of self-pride or emotions may be discouraged or interpreted as inconsistent with cultural expectations of humility. Consequently, adolescents may be less likely to strongly endorse these items, as personal achievements are often viewed as collective or family successes rather than individual accomplishments. Thus, the weaker loading of this item likely represents cultural variation in the expression of self-efficacy and resilience rather than a psychometric limitation of the scale.

For the CYRM-R, while the original two-factor structure, distinguishing *personal* and *relational resilience* [27], demonstrated reasonable model fit, the exploratory and confirmatory analyses in the Nepali adolescent sample also suggested a two-factor model alongside a coherent representation through a unidimensional structure. However, in contrast to the original CYRM-R structure, several family and caregiver support items loaded on the personal resilience factor among Nepali adolescents. This may reflect the strong collectivist orientation in Nepal, where the “self” is relational and embedded within family systems. As such, family support, protection, and provision are interpreted not as external social support but as core components of an adolescent’s intra-personal strength and identity. Conversely, items reflecting peer, school, and community support loaded onto the relational factor. In the Nepali context, these systems are experienced as more variable and external, and therefore represent social opportunities rather than personal capacities. Moreover, a strong positive standardized correlation was observed between the two factors, suggesting substantial overlap between the constructs, although they remain theoretically distinct based on the pattern of factor loadings. These culturally grounded interpretations likely shaped the observed factor structure of the CYRM-R in Nepali adolescents, suggesting meaningful contextual differences in how resilience is conceptualized. Furthermore, the CYRM-R item 2 (*Getting an education is important to me*) exhibited lowest/modest loadings in both the one- and two-factor solutions, indicating anomaly attributed to various socio-cultural and contextual factors specific to Nepal, leading to divergence in interpreting the item. Educational aspirations in Nepal are shaped by socioeconomic factors and family expectations [47], such as those facing barriers like financial constraints and household responsibilities, leading to varied perceptions of education’s importance and its alignment with resilience measure. The number of factors found in the Indian population was a 3-factor (individual, relational, and contextual) model with the items loading in a different way [55]. This finding is similar to the factorial structure found for the CYRM versions in the Spanish [52] and Brazilian populations [53]. These findings are logical, as the socio-ecological framework of resilience emphasizes the significance of culture and context [54]. This pattern may reflect the interconnected nature of personal and relational resources in collectivist settings such as Nepal, where individual coping and external supports are closely inter-twined. The one-factor model, which demonstrated acceptable fit indices, therefore provides a parsimonious and conceptually meaningful representation of resilience as an integrated construct encompassing both personal strengths and relational supports within the Nepali cultural context. Despite having modest factor loadings for some items (such as item 2, item 3) the decision to retain all items within the final Nepali version of the CYRM-R was also aided by the good communalities for most of the items and good internal consistency for the total scale as well as subscales in the Nepalese sample, as suggested by the literature for retaining items in the tool [56].

The measurement invariance analyses indicate that both the CYRM-R, including the 1-factor and 2-factor models, and RRM demonstrate largely comparable factor structures across male and female adolescents, suggesting that the overall resilience construct is interpreted similarly across genders. This finding validates the use of both measures for cross-gender comparisons, confirming that the latent constructs of external and internal resilience are broadly equivalent for male and female adolescents in the Nepali context.

Preliminary validity analyses provided support for both convergent and known-groups validity of the RRM and CYRM-R scales and sub-scales (personal and relational resilience) in Nepali adolescents. The strong positive correlation between the CYRM-R and RRM indicates that internal resilience assets and external resilience resources are closely related, while the negative associations with depressive and anxiety symptoms align with theoretical expectations. Examination of known-groups validity showed no gender differences on the resilience measures. This pattern may reflect that both the CYRM-R and RRM are well adapted measures to function and examine resilience equivalently across male and female adolescents in Nepali context.

The preliminary results in the current study showed moderate test-retest reliability of the scales and sub-scales of the resilience measures. There was a similar result for the 2-week test-retest reliability of CYRM-28 among French Canadian youths, with the test-retest correlation coefficient values above 0.70. for total as well as sub-scales [57]. Overall, the findings of the present study suggest that tailoring CYRM-R and RRM for Nepalese adolescents provided culturally sensitive, contextually relevant and psychometrically sound resilience instruments, allowing for a more comprehensive and systemic approach encompassing the protective factors from both internal and external resource perspectives.

There are three limitations of this study. The study focused on adolescents experiencing urban poverty, which may limit its generalizability to rural populations facing different adversities or those not living in poverty. A key consideration of the content validation is that expert ratings were collected retrospectively (post-hoc) rather than before data collection. While retrospective ratings can potentially introduce bias, in this case, the experts did not suggest major changes, and the results indicate good content validity. Nevertheless, future studies could benefit from prospective content validation with independent experts before large-scale administration to further strengthen confidence in the measures. Finally, we recommend that future research further examine the test–retest reliability of these resilience measures, as the small sample size in the present study limits the precision of these estimates; thus, the current findings should be considered preliminary.

Using the Nepali versions of CYRM-R and RRM together can help researchers, educators, and mental health practitioners identify protective factors, design effective interventions, and support adolescents facing adversity. Furthermore, the findings also contribute to global discussions on resilience by incorporating perspectives from the global south, enriching the understanding of how resilience is operationalized in diverse cultural landscapes. Adapting these tools is, therefore, a critical step in promoting adolescent well-being and fostering their potential to thrive in Nepal’s complex socio-cultural environment.

For future research, it is essential to examine how CYRM-R and RRM perform in capturing both internal and external protective factors within Nepal’s unique cultural and socio-ecological environment. Studies should explore how these resilience measures work in line with other resilience measures to establish criterion validity. Similarly, future research should examine how resilience differs across Nepalese subpopulations, particularly among marginalised caste groups who may experience unique stressors.

## Conclusion

Following a rigorous multi-step translation and adaptation process, the Nepali versions of the CYRM-R and RRM have demonstrated good validity and reliability. Based on data from the Nepalese sample, the CYRM-R is well-structured around two key latent factors (personal and relational), with only modest cross-loadings for a few items, and also demonstrated acceptable reasonable unidimensionality; thus, it is recommended for use both as a unidimensional measure to examine overall score as well as two-dimensional factor of external resilience to explore personal and relational resilience. The RRM, in contrast, is supported as a robust single-factor measure. These models have been validated, confirming that the CYRM-R measures resilience within a social and environmental context, while the RRM assesses resilience based on internal psychological strengths. Similarly, good internal consistency and preliminary acceptable test-retest results indicate the reliability of these instruments in Nepalese contexts. Both tools are well-suited for assessing resilience among Nepalese adolescents at the population level, for both males and females. Researchers and practitioners are encouraged to utilize these measures to gain a comprehensive understanding of resilience, encompassing both internal and external resources.

## Supplementary Material

The online version contains supplementary material available at https://doi.org/10.1007/s10578-025-01944-x.

Supplementary Files

## Figures and Tables

**Table 1:** Descriptive statistics of the items of CYRM-R and RRM

Tool & Item No.	Item	Mean (SD)	Skewness (SE)	Kurtosis (SE)	Polychoric/ Ordinal ITC
CYRM-R					
CYRM-R1	I get along with people around me	3.93 (0.95)	-1.21 (0.31)	1.83 (0.60)	0.31
CYRM-R2	Getting an education is important to me	4.44 (0.76)	-1.65 (0.31)	3.07 (0.60)	0.20
CYRM-R3	I know how to behave in different social situations	4.05 (0.76)	-0.55 (0.31)	0.19 (0.60)	0.26
CYRM-R4	My parent(s)/caregiver(s) really look out for me	4.28 (1.04)	-1.43 (0.31)	1.23 (0.60)	0.40
CYRM-R5	My parent(s)/caregiver(s) know a lot about me	3.36 (1.11)	-0.62 (0.31)	0.01 (0.60)	0.35
CYRM-R6	If I am hungry, there is enough to eat	3.34 (1.12)	-0.29 (0.31)	-0.79 (0.60)	0.31
CYRM-R7	People like to spend time with me	3.70 (0.92)	-0.70 (0.31)	0.35 (0.60)	0.32
CYRM-R8	I talk to my family/caregiver(s) about how I feel	2.80 (1.38)	0.21 (0.31)	-1.21 (0.60)	0.28
CYRM-R9	I feel supported by my friends	3.52 (0.98)	-0.68 (0.31)	0.09 (0.60)	0.32
CYRM-R10	I feel that I belong/belonged at my school	4.16 (0.78)	-0.52 (0.31)	-0.48 (0.60)	0.39
CYRM-R11	My family/caregiver(s) stand by me during difficult times	4.08 (0.97)	-0.85 (0.31)	-0.23 (0.60)	0.34
CYRM-R12	My friends stand by me during difficult times	3.61 (1.04)	-0.62 (0.31)	-0.14 (0.60)	0.33
CYRM-R13	I am treated fairly in my community	3.67 (0.91)	-0.40 (0.31)	0.12 (0.60)	0.30
CYRM-R14	I have opportunities to show others that I am becoming an adult and can act responsibly	3.87 (1.01)	-0.84 (0.31)	0.16 (0.60)	0.27
CYRM-R15	I feel safe when I am with my family/caregiver(s)	4.31 (0.96)	-1.73 (0.31)	3.28 (0.60)	0.38
CYRM-R16	I have opportunities to develop skills that will be useful later in life (like job skills and skills to care for others)	3.89 (0.97)	-0.90 (0.31)	1.02 (0.60)	0.33
CYRM-R17	I enjoy my family’s/caregiver’s cultural and family traditions	4.34 (0.89)	-1.33 (0.31)	1.02 (0.60)	0.33
RRM					
RRM1	I believe in myself	3.94 (0.89)	-0.61 (0.30)	-0.19 (0.60)	-0.07
RRM2	I can adapt to challenging situations	3.44 (1.02)	-0.20 (0.30)	-0.75 (0.60)	-0.02
RRM3	I find solutions to problems I encounter	3.44 (0.95)	-0.05 (0.30)	-0.91 (0.60)	0.13
RRM4	I can keep going despite difficulties	3.90 (0.84)	-0.66 (0.30)	1.01 (0.60)	0.54
RRM5	I can cope with competing demands (for my time or attention)	3.74 (0.92)	-0.36 (0.30)	-0.61 (0.60)	-0.17
RRM6	Even when there are setbacks or obstacles, I am hopeful about my future	4.26 (0.81)	-1.09 (0.30)	1.03 (0.60)	-0.07
RRM7	I am generally in control of my emotions	3.37 (0.98)	0.05 (0.30)	-0.53 (0.60)	-0.64
RRM8	I take pride in things I have achieved	4.34 (0.77)	-1.12 (0.30)	1.13 (0.60)	-0.77
RRM9	When faced with difficulties, I rise to the challenge	3.44 (1.11)	-0.35 (0.30)	-0.58 (0.60)	0.34
RRM10	I can find meaning in my life	3.65 (0.91)	-0.86 (0.30)	1.05 (0.60)	-0.15

**Table 2:** Internal consistency of scales and subscales of Nepali versions of resilience instruments

Resilience scales/Subscales	Polychoric Mean Inter-Item Correlations	Cronbach’s Alpha α (Ordinal Alpha)	McDonald’s Omega Ω
CYRM-R scale (all 17 items) (EFA Sample)	0.32	0.89	0.91
CYRM-R scale (all 17 items) (CFA Sample)	0.28	0.87	0.84
Personal Resilience (CYRM-R items 1,2,3,4,6,11,14,15,16,17) (EFA Sample)	0.33	0.83	0.86
Personal Resilience (CYRM-R items 1,2,3,4,6,11,14,15,16,17) (CFA Sample)	0.27	0.78	0.73
Relational Resilience (CYRM-R items 5,7,8,9,10,12,13) (EFA Sample)	0.39	0.82	0.81
Relational Resilience (CYRM-R items 5,7,8,9,10,12,13) (CFA Sample)	0.33	0.77	0.75
RRM scale (all 10 items) (EFA Sample)	0.39	0.86	0.86
RRM scale (all 10 items) (CFA Sample)	0.34	0.84	0.81

**Table 3:** Factor loadings of One- and Two-Factor solutions of CYRM-R and One-Factor solution of RRM Nepali items for PAF with oblimin rotation

Tool/Item	1 Factor Model		2 Factor Model		
	Factor Loading	Communalities	Factor 1 Loading	Factor 2 Loading	Communalities
CYRM-R1	0.53	0.28	**0.31**	0.28	0.28
CYRM-R2	0.35	0.12	**0.28**	0.10	0.12
CYRM-R3	0.46	0.21	**0.35**	0.16	0.21
CYRM-R4	0.71	0.51	**0.80**	-0.01	0.63
CYRM-R5	0.62	0.38	0.30	**0.40**	0.39
CYRM-R6	0.55	0.30	**0.32**	0.30	0.30
CYRM-R7	0.56	0.31	0.19	**0.44**	0.33
CYRM-R8	0.50	0.25	0.20	**0.36**	0.26
CYRM-R9	0.57	0.32	-0.12	**0.80**	0.54
CYRM-R10	0.69	0.47	0.21	**0.57**	0.51
CYRM-R11	0.63	0.40	**0.49**	0.21	0.41
CYRM-R12	0.59	0.35	0.02	**0.67**	0.47
CYRM-R13	0.52	0.27	0.01	**0.59**	0.35
CYRM-R14	0.49	0.24	**0.45**	0.08	0.26
CYRM-R15	0.68	0.46	**0.82**	-0.07	0.61
CYRM-R16	0.59	0.35	**0.67**	-0.01	0.43
CYRM-R17	0.59	0.35	**0.55**	0.10	0.38
RRM1	0.64	0.42	-	-	-
RRM2	0.63	0.39	-	-	-
RRM3	0.66	0.44	-	-	-
RRM4	0.77	0.59	-	-	-
RRM5	0.61	0.38	-	-	-
RRM6	0.64	0.41	-	-	-
RRM7	0.48	0.23	-	-	-
RRM8	0.43	0.18	-	-	-
RRM9	0.74	0.54	-	-	-
RRM10	0.60	0.36	-	-	-

Note: Factor correlations between the 2 factors of CYRM-R were 0.59

**Table 4:** CFA fit indices for one factor model for Nepali RRM and one- and Two-Factor model for CYRM-R

Indices	CYRM-*R* (1-Factor)	CYRM-*R* (2-Factor)	RRM (1-Factor)
xW	2.259	1.797	1.108
TLI (robust)	0.735	0.757	0.932
CFI (robust)	0.768	0.790	0.947
RMSEA (robust)	0.098	0.094	0.064
SRMR	0.079	0.078	0.053

Note: Good/Acceptable fit thresholds: Chi-Square Minimum/degree of freedom (χ^2^/df) ≤ 3, Tucker_Lewis Index (TLI)/Comparative Fit Index (CFI) ≥ 0.90, Root Mean Square Error of Approximation (RMSEA)/Standardized Root Mean Square Residual (SRMR) ≤ 0.08; Factor Covariance CYRM-R: F1-F2 = 0.36 and Correlation = 0.85

**Table 5:** Bivariate (Spearman’s) correlations between variables

Variables	Poverty	PHQA	GAD7	CYRM-*R*	PR	RR	RRM
Poverty	-						
PHQA	0.064	-					
GAD7	0.103*	0.735**	-				
CYRM-R	-0.123**	-0.260**	-0.222**	-			
PR	-0.090*	-0.211**	-0.193**	0.899**	-		
RR	-0.134**	-0.256**	-0.215**	0.898**	0.627**	-	
RRM	-0.091*	-0.223**	-0.189**	0.676**	0.655**	0.567**	-

Note: *Correlation significant at 0.05 level, **Correlation significant at 0.001 level; PHQA: depression score; GAD7: anxiety score; CYRM-R: external resilience score; PR/RR: 2 factors of CYRM-R representing personal and relational resilience score; RRM: internal resilience score

**Table 6:** Preliminary ICC for Nepali versions of CYRM-R and RRM (*n* = 20)

Domain/Scale/Tool	ICC	95% Confidence Interval of ICC	ICC & 95% ICC	
			Male *(n* = 10)	Female (*n* = 10)
CYRM-R scale (all items)	0.69	0.20, 0.88	0.48 (-1.01, 0.87)	0.76 (-0.03, 0.94)
-Personal Resilience	0.61	0.01, 0.85	0.27 (-1.69, 0.82)	0.74 (-0.13, 0.94)
-Relational Resilience	0.73	0.30, 0.89	0.81 (0.21, 0.95)	0.68 (-0.46, 0.92)
RRM scale	0.70	0.15, 0.89	0.58 (-0.28, 0.89)	0.83 (0.35, 0.96)

## Data Availability

All the essential data used are presented within the manuscript. R.S., M.J.D.J., and C.L. confirm that they had full access to all the data in the study and take responsibility for the integrity of the data and the accuracy of the data analysis. Interested parties may notify the investigators of their interest in collaboration, including access to the data set analyzed here, through the following rakesh.singh@kcl.ac.uk or rakes4r@gmail.com.
