# Supplementary material for: Translation and Adaptation of the Child and Youth Resilience Measure-Revised and Rugged Resilience Measure: A Mixed-Method Study Among Adolescents in Nepal
Source: Child Psychiatry Hum Dev. Author manuscript; Available in PMC 2026 Aug 10. (PMC7619341; doi:10.1007/s10578-025-01944-x)
Supplement: Supplementary file 1 [file EMS217419-supplement-Supplementary_Files.docx]

**Table S1.** STROBE Statement—Checklist of items that should be included in reports of cross-sectional studies

|  | Item No | Recommendation |  | Page No. |  |  |  |  |  |  |
| --- | --- | --- | --- | --- | --- | --- | --- | --- | --- | --- |
| Title and abstract | 1 | (*a*) Indicate the study’s design with a commonly used term in the title or the abstract |  | 1 |  |  |  |  |  |  |
|  |  | (*b*) Provide in the abstract an informative and balanced summary of what was done and what was found |  | 3 |  |  |  |  |  |  |
| Introduction | | |  |  |  |  |  |  |  |  |
| Background/rationale | 2 | Explain the scientific background and rationale for the investigation being reported |  | 5-7 |  |  |  |  |  |  |
| Objectives | 3 | State specific objectives, including any prespecified hypotheses |  | 7 |  |  |  |  |  |  |
| Methods | | |  |  |  |  |  |  |  |  |
| Study design | 4 | Present key elements of study design early in the paper |  | 7 |  |  |  |  |  |  |
| Setting | 5 | Describe the setting, locations, and relevant dates, including periods of recruitment, exposure, follow-up, and data collection |  | 7-8 |  |  |  |  |  |  |
| Participants | 6 | (*a*) Give the eligibility criteria, and the sources and methods of selection of participants |  | 7-8 |  |  |  |  |  |  |
| Variables | 7 | Clearly define all outcomes, exposures, predictors, potential confounders, and effect modifiers. Give diagnostic criteria, if applicable |  | N/A |  |  |  |  |  |  |
| Data sources/ measurement | 8* | For each variable of interest, give sources of data and details of methods of assessment (measurement). Describe comparability of assessment methods if there is more than one group |  | 7-10 |  |  |  |  |  |  |
| Bias | 9 | Describe any efforts to address potential sources of bias |  | 8-15 |  |  |  |  |  |  |
| Study size | 10 | Explain how the study size was arrived at |  | 7-9 |  |  |  |  |  |  |
| Quantitative variables | 11 | Explain how quantitative variables were handled in the analyses. If applicable, describe which groupings were chosen and why |  | 13-14 |  |  |  |  |  |  |
| Statistical methods | 12 | (*a*) Describe all statistical methods, including those used to control for confounding |  | 13-14 |  |  |  |  |  |  |
|  |  | (*b*) Describe any methods used to examine subgroups and interactions |  | 13-14 |  |  |  |  |  |  |
|  |  | (*c*) Explain how missing data were addressed |  | N/A |  |  |  |  |  |  |
|  |  | (*d*) If applicable, describe analytical methods taking account of sampling strategy |  | N/A |  |  |  |  |  |  |
|  |  | (*e*) Describe any sensitivity analyses |  | N/A |  |  |  |  |  |  |
| Results | | |  |  |  |  |  |  |  |  |
| Participants | 13* | (a) Report numbers of individuals at each stage of study—eg numbers potentially eligible, examined for eligibility, confirmed eligible, included in the study, completing follow-up, and analysed |  | 8-14 |  |  |  |  |  |  |
|  |  | (b) Give reasons for non-participation at each stage |  | N/A |  |  |  |  |  |  |
|  |  | (c) Consider use of a flow diagram |  | N/A |  |  |  |  |  |  |
| Descriptive data | 14* | (a) Give characteristics of study participants (eg demographic, clinical, social) and information on exposures and potential confounders |  | 7 |  |  |  |  |  |  |
|  |  | (b) Indicate number of participants with missing data for each variable of interest |  | N/A |  |  |  |  |  |  |
| Outcome data | 15* | Report numbers of outcome events or summary measures |  | N/A |  |  |  |  |  |  |
| Main results | 16 | (*a*) Give unadjusted estimates and, if applicable, confounder-adjusted estimates and their precision (eg, 95% confidence interval). Make clear which confounders were adjusted for and why they were included |  | 15-31 |  |  |  |  |  |  |
|  |  | (*b*) Report category boundaries when continuous variables were categorized |  | 15-31 |  |  |  |  |  |  |
|  |  | (*c*) If relevant, consider translating estimates of relative risk into absolute risk for a meaningful time period |  | N/A |  |  |  |  |  |  |
| Other analyses | 17 | Report other analyses done—eg analyses of subgroups and interactions, and sensitivity analyses |  | 27-31 |  |  |  |  |  |  |
| Discussion | | |  |  |  |  |  |  |  |  |
| Key results | 18 | Summarise key results with reference to study objectives |  | 31-33 |  |  |  |  |  |  |
| Limitations | 19 | Discuss limitations of the study, taking into account sources of potential bias or imprecision. Discuss both direction and magnitude of any potential bias |  | 36-37 |  |  |  |  |  |  |
| Interpretation | 20 | Give a cautious overall interpretation of results considering objectives, limitations, multiplicity of analyses, results from similar studies, and other relevant evidence |  | 32-36 |  |  |  |  |  |  |
| Generalisability | 21 | Discuss the generalisability (external validity) of the study results |  | 37-38 |  |  |  |  |  |  |
| Other information | | |  |  |  |  |  |  |  |  |
| Funding | 22 | Give the source of funding and the role of the funders for the present study and, if applicable, for the original study on which the present article is based |  | 7 |  |  |  |  |  |  |

*Give information separately for exposed and unexposed groups.

Note: Information on the STROBE Initiative is available at www.strobe-statement.org.

**Table S2.** Participant characteristics

| Characteristics | Categories | n (%) or mean (SD) |
| --- | --- | --- |
| Participants in the pilot assessment |  |  |
| Age^#^ | N/A | 13.35 (1.29) |
| Grades |  |  |
|  | Six | 22 (35.5%) |
|  | Seven | 16 (25.8%) |
|  | Eight | 24 (38.7%) |
| Gender |  |  |
|  | Male | 29 (46.8%) |
|  | Female | 33 (53.2%) |
| Ethnicity |  |  |
|  | Janajati | 26 (41.9%) |
|  | Brahim/Chhetri | 14 (22.6%) |
|  | Dalit | 13 (21%) |
|  | Others | 9 (14.5%) |
| Participants in the survey |  |  |
| Age^#^ | N/A | 13.85 (0.73) |
| Grades |  |  |
|  | Six | 109 (22.2%) |
|  | Seven | 210 (42.9%) |
|  | Eight | 171 (34.9%) |
| Gender |  |  |
|  | Male | 232 (47.3%) |
|  | Female | 258 (52.7%) |
| Ethnicity |  |  |
|  | Janajati | 247 (50.4%) |
|  | Brahim/Chhetri | 134 (27.4%) |
|  | Dalit | 51 (10.4%) |
|  | Madhesi | 37 (7.6%) |
|  | Muslim | 20 (4.1%) |

Note: ^#^ refers to Mean (SD), N/A refers to Not Applicable

**Table S3.** Item-wise adaptations for CYRM-R

| Item # CYRM-R | Original CYRM-R Item wording | Nepali version | Nepali English Version | Findings from FGDs | Findings from CIs |
| --- | --- | --- | --- | --- | --- |
| CYRM-R1 | I get along with people around me | तिम्रो वरपर भएका मानिसहरु (जस्तै साथीभाई, आफन्त, छरछिमेक) संग तिमी कत्तिको मिलेर बस्छौ ? (मिलेर बस्नु भनेको जस्तै एक अर्कोलाई सहयोग गर्ने, खाने, हिड्ने, खेल्ने, काम गर्ने आदि) | How much can you stay together in harmony with people around you (For example, friends, relatives, neighbors)  (Staying together in harmony would mean helping each other, eating, playing, working together, etc) | Most of the adolescents of all groups understood this item. They related *staying together* to get *along with each other* and gave examples of *staying together* such as helping others during community work and school activities. While some adolescents said they would do the activities at the school and community level by helping each other like plantation of the trees, others said, getting along means helping each other and staying together in place. They also said that instead of getting along, staying together is a better word. However, some adolescents in a few groups had some problems understanding the phrase “staying together”, the examples of staying together in harmony in the bracket were added. Similarly, to understand this question some of the groups suggested adding examples of people like friends, relatives, and neighbors. | While some of the adolescents linked this item only with staying together, others related with helping each other when individuals need support.  A girl aged 14 years said, “I only do a little work with the help of friends because all work can’t be completed only with the support of friends. I do my work myself and only take support for those works which can’t be done alone. In this question, I would choose the option “a little bit” because I have a few works that I can’t do by myself and will take support from others.  Similarly, a boy aged 13 years said, it is obvious that when I need help my friends will come and help me. Likewise, when my friends are in trouble, I will go and help them. We help each other not only at difficult times but also at other times such as while doing project work at school. |
| CYRMR-2 | Getting an education is important to me | तिम्रो लागि पढाई लेखाई कत्तिको महत्वपुर्ण छ ? | How important is education for you? | All groups understood this item clearly. Some of the groups suggested replacing education (*shikshya*) with *padhaai lekhaai* in Nepali version so that everyone enrolled at public school *can understand the* item easily.  The groups said, if we study, we will get a better job in the future, our life will be settled, and don’t have to face any difficulties. Not only this education helps us to go for foreign employment as well as to become a successful person. | All adolescents understood this item clearly without confusion and shared their experiences.  A younger boy aged 13 years chose the option “a lot” for this item and said that in today’s generation, all people need education and without education, they can’t do anything. Even money can’t do anything for us. If I have an education I can get a job at a higher level, I can start up my own business, and no need to go for foreign employment.  Similarly, a 15-year-old girl said, I have to study and settle my life as well as have to take care of my parents. If I didn’t study then I can’t do anything else. That’s why, education is very important to me. |
| CYRM-R3 | I know how to behave in different social situations | तिमीलाई विभिन्न स्थानहरु (जस्तै स्कुल, मन्दिर, घर, आदि) मा कसरी व्यवहार गर्ने भन्ने कत्तिको थाहा छ ? | How much do you know how to behave in different places (For example, school, temple, house, etc) ? | All groups understood this item, however, a few of the participants suggested replacing the words “community activities” with “places” so that it would be easy for them to describe behavior in specific places such as home, school, and temple. This was in line with the understanding that some of the boys understood the phrase “community activities” as parties and political programs. The groups said this item is asking whether we know to show different behaviors in various situations or not such as being polite, helping each other, showing respect, knowing how to talk, sit, behave, and maintain discipline, or not. | The adolescents understood this item and linked with their personal experiences.  Adolescent aged 13 years said, when we go to different places such as at school, we have to respect teachers and talk politely, with friends we have to help and cooperate and have to behave well when we see new people at the temple. A boy shared his experiences, I had gone to new places and I met people of an old age and I did *Namaskar* to them. Likewise, I showed love to the junior ones. However, a 15-year-old adolescent said, only to some extent I know because I may not know how to behave in different places all the time. Sometimes I don’t know and have to learn from other people’s behavior. |
| CYRM-R4 | My parent(s)/caregiver(s) really look out for me | तिम्रो अभिभावकले तिमीलाई साँच्चिकै हेरबिचार गर्नुहुन्छ जस्तो कत्तिको लाग्छ ? | How much do you feel like your parents really look after you? | All groups understood this item.  The adolescents said this question is saying whether our parents loved, took care or looked after us or not, whether we studied or not. | All adolescents understood this question and linked it with their experiences.  An adolescent aged 14 years responded I would say a lot as a response to this item because I lived with my maternal uncle and when I reached home later than the usual time, he started getting scared. They take care of me where I am going and what am I doing.  Similarly, a 15-year-old girl said I would say to some extent only because it’s due to my family problem. In my family, we are only two kids of our parents and I am the elder one. My parent takes care of my brother as he is the younger one and he needs more love and care. I had grown up and I could stand on my own feet too. |
| CYRM-R5 | My parent(s)/caregiver(s) know a lot about me | तिम्रो अभिभावकहरुलाई तिम्रो बारेमा धेरै नै थाहा छ जस्तो कत्तिको लाग्छ ? (जस्तै तिम्रो साथीहरु को को हुन्, तिमीलाई के गर्न मन लाग्छ, आदि) | How much do you feel like your parents know a lot about you? (For example, who your friends are, what you want to do, etc) | All groups easily understood the item.  Most of the adolescents in all groups described that their parents knew a lot about them and related the phrase “knowing a lot” with examples such as behaviors, good/bad habits as well as friends circle.  The groups said this item means whether our parents know about us or our secrets. Knowing a lot means knowing our friends where he/she lives, where we are going, what’s our education level, and how we behave with others. | All adolescents were able to link the item with their experiences.  A younger adolescent said my mother knows a lot about me because she loves hanging out with my friends. I share everything with my mummy. If I don’t share also, she understands by looking at my face and my behavior.  Likewise, another 14-year-old adolescent said, to some extent only they know because everything can’t be shared with the parents. Only we let the parents know about our good things. But my parents keep on trying to know everything from my friends and sometimes they knew from my behaviors too. |
| CYRM-R6 | If I am hungry, there is enough to eat | तिमीलाई भोक लागेको बेलामा खानलाई घरमा टन्न खानेकुराहरु हुन्छ जस्तो कत्तिको लाग्छ ? | How much do you feel like there’s a lot of food at home for you to eat when you are hungry? | The groups understood the item. However, there was a bit of confusion among some adolescents in a few groups regarding the scenario of the question and suggested adding the name of place “home” within the item. They rephrased the item themselves into, *how much do you feel there’s a lot of food to eat at home when you are hungry?*  The groups understood that when they are hungry, whether there’s the availability of food at their home or not. | All adolescents understood the item related to their personal experiences.  A 14-year-old boy said at my house I get whatever I want to eat. When we were in the village, it was difficult to get food and there was not so much food in my kitchen.  A younger adolescent aged 13 years said I get food whenever I want to eat. When I have to eat breakfast, I take out snacks from the kitchen and eat. That’s why I would choose a lot for this item because my mother keeps everything that I want to have and if some food gets finished, she brings it from the market immediately. Also, she says, if you want to eat something, tell me I will bring and I tell her accordingly, and she will bring it. |
| CYRM-R7 | People like to spend time with me | अरु मानिसहरु तिमीसँग समय बिताउन कत्तिको रमाइलो मान्छन् ? (मानिसहरु जस्तै साथीहरु, दाजुभाई, दिदीबहिनी, आदि) | How much do other people like spending time with you? (Other people like friends, siblings, etc) | All groups understood the item, however, they suggested having the phrase “other people” in place of people and adding examples of people in brackets.  The groups said this item is asking whether your friends, brothers, sisters, or relatives like to talk, want to have fun with, or like to spend time with us or not. Spending time means playing, sitting, chatting, and sharing things.  The adolescents also related the item by saying if our friends don’t want to spend time with them, they will make an excuse otherwise they love to spend time playing, talking, and sharing things. | All adolescents understood the item and linked it with their experiences.  An older respondent aged 16 years said, the people like me a lot because I speak well and cooperate with the children. Not only that, I make them laugh by telling jokes. So, children love to spend time with me.  Likewise, a younger adolescent aged 13 years said I would respond “to some extent only” to this question. As I am the youngest son in my family, my brother and sister come to play, laugh, and speak with me. No other member of the family is like me. People have different ways of having fun such as some have by playing with friends, and some by laughing. |
| CYRM-R8 | I talk to my family/caregiver(s) about how I feel | तिमी आफ्ना feelings (जस्तै दु:ख, खुशी, डर, चिन्ता, रिस, आदि) को बारेमा आफ्नो परिवारसँग कत्तिको कुरा गर्छौ ? | How much do you talk about your feelings (For example, sadness, happiness, fear, worries, anger, etc) with your family ? | Almost half of the groups had difficulty understanding the item, particularly the term “feelings” and after adding examples of the feelings such as sadness, happiness, fear, worries, anger, etc. in the bracket, the item was easily understood by all groups.  The groups said the item  is asking how often we share feelings such as fear or sadness with our parents. I talk with my sisters or brothers, *mummy or daddy* when I have such feelings.  Similarly, an adolescent within a group shared her experience, “one day she felt bad at school and she shared it with her *mummy* which made her feel good. But sometimes when my parents ignored her feelings when she opened up then she felt like it was pointless to speak up”. | All adolescents linked the item with their personal experiences.  Most of the participants shared their feelings with their mothers.  An 11-year-old adolescent said, I only talk about my happy feelings, not for feelings like fear, anger, and worries. That’s why I would choose to some extent only because I like to share only happy moments such as if some fun moments happened at school, then only, I would share otherwise I am not interested in sharing other feelings.  Similarly, another adolescent aged 15 years said, I share both happy and sad feelings with my parents. I share what I feel then only, it can be resolved as I share my problems with those people who can resolve them. Mostly, I share with my parents in the family. |
| CYRM-R9 | I feel supported by my friends | तिमीलाई तिम्रो साथीहरुले मद्दत गर्छन् जस्तो कत्तिको लाग्छ ? | How much do you feel like your friends help you? | All groups understood the item. However, some adolescents in a few groups suggested replacing the word *sahayog* with *maddat.*  The groups linked the item to their daily lives and said the item is asking how much their friends help them while doing homework or to get rid of difficult situations. They said “helping each other” means sharing food between friends when they forget to bring snacks, helping to solve problems, and so on. | All adolescents were able to link this item with their personal experiences.  An adolescent aged 16-year-old said my friends help me when I need help because I have also helped them. We can support others when they are in trouble but at other times, they can do it themselves. Sometimes my friends can encounter a problem such as girls who are having periods, I can help them by buying sanitary pads. Similarly, for boys, I can help when they have an accident.  The younger adolescent aged 12 years said, my friends help me a lot because sometimes I forget to do my homework or to bring a copy at that time my friends help in doing homework. My friends also help me in situations like sometimes I forget to bring tiffin from my home and have to stay at tuition until 7 pm, that time if my other friends have money, he/she share with me. In this way my friends support me. |
| CYRM-R10 | I feel that I belong/belonged at my school | तिमीलाई आफु पढ्ने विद्यालय आफ्नै जस्तो कत्तिको लाग्छ ? (आफ्नै जस्तो भन्नाले शिक्षकहरुले friendly तरिकाले पढाउने ,सिकाउने,साथीहरुसंग मिलेर बस्ने, एकअर्कालाई सहयोग गर्ने आदि ) | How much do you feel like the school where you study is like your own? (For example, teachers teaching and guiding you in a friendly manner, getting along together with friends, helping each other, etc) | The groups did not understand the term “belong at” and suggested replacing them with “like your won (*aafnai jaasto)”.* They understood belonging as looking after the school, managing waste, and conducting programs. After the suggested adaptation was considered, the groups understood the item and said that the item asked whether they felt their school environment being friendly such as helping each other, understanding our feelings by the teacher, showing love and care by the teacher as well as sharing thoughts.  One of the adolescents in one group suggested changing the translations to, “How much do you feel like your school is like your home?” However, this was not recommended by others as “like home” would mean differently and could lead to wrong interpretation. | All adolescents linked this item with their experiences and said related their schools with their house by saying, “*aafno ghar jastai laagcha.”*  An adolescent aged 16 years responded “a lot” to the item and said at my school my friends and teachers are like my parents and sisters. My first parent is my home whereas my second parent is my teachers.  Likewise, the younger adolesent aged 11 years said, the teacher must be friendly with the students. For this item, I chose a lot because the teachers are close with the students and they treat students like their children, brothers as well as sisters. School is also like a home where they teach us to move ahead on the right track which is the same as parents. |
| CYRM-R11 | My family/caregiver(s) stand by me during difficult times | तिम्रो परिवारले तिमीलाई अप्ठ्यारो परेको बेलामा (जस्तै बिरामी हुँदा, काम बिगार्दा, आदि) साथ दिनुहुन्छ जस्तो कत्तिको लाग्छ ? | How much do you feel like your family will support you when you are in a difficult situation (For example, when you are sick, when you do something wrong, etc)? | All groups understood this item.  The groups said the question is asking how much our family members support, care, and understand our problems when we are at school, at home, or face any difficult situations such as looking after when we have a headache or suffering from any health problems. | All adolescents linked the item with their experiences and said they were supported by their parents in difficult situations.  A young adolescent aged 11 years said, when I am feeling unwell my *mummy* understands and brings medicine along with hot water for me. She never leaves me alone when I am sick. She supports me in every step. One day my friend blamed me for breaking the windows but it was by my friend. I shared with my *daddy*. That time he supported me and told me not to be scared if you are not doing wrong.  Likewise, another adolescent responded “a lot” because my parents solved any problem that I faced. One day I beat my friend and my father knew about it. That time also he counseled me not to repeat such kinds of mistakes. |
| CYRM-R12 | My friends stand by me during difficult times | तिम्रो साथीहरुले तिमीलाई अप्ठ्यारो परेको बेलामा (जस्तै बिरामी हुँदा, काम बिगार्दा, आदि) सहयोग गर्छन् जस्तो कत्तिको लाग्छ ? | How much do you feel like your friends will help you when you are in a difficult situation (For example, when you are sick, when you do something wrong, etc)? | All groups understood the item. However, the groups suggested replacing *saath with sahayog* for “stand by”.  The groups said the item is about supporting each other between friends in difficult situations, for example, getting help to do homework when we face difficulties or dropping a friend at home when feeling sick. | All adolescents linked the item with their personal experiences.  The younger participant aged 12 years said one day I had a headache and was unable to sit in the classroom and my friends dropped me off at home. Also, sometimes I made a mistake by myself but my friend forgave me too.  Another adolescent aged 15 years said, my friends support me more than I need. One day I made a mistake by myself and I blamed my friend but he accepted the mistake that was made by me. I am friendly with my friends; talk politely as well I am good at studying. That’s why my friend helps me in difficult times. |
| CYRM-R13 | I am treated fairly in my community | तिम्रो समुदायका मानिसहरुले तिमी सँग राम्रो व्यवहार गर्छन् जस्तो कत्तिको लाग्छ ? (राम्रो व्यवहार भन्नाले सानोलाई माया गर्ने, झुट कहिले नबोल्ने,चोरी नगर्ने, मिठो बोल्ने आदि) | How much do you feel like the people in your community show good behavior towards you? (Good behavior as in loving those younger than you, never lying to you, never stealing from you, talking nicely to you, etc) | Most of the groups did not understand what exactly is “fair treatment /equal behavior (*uchit vyabahaar)"* and preferred replacing them with “good behavior *(ramro vyabahaar)*. They described good behavior as treating equally to all people or helping people when needed. They also suggested replacing “community” by “community people”.  The groups said this question is asking about how community people behave with us. Good behavior means speaking politely to us, supporting us when we face problems, respecting seniors, and loving juniors as well as helping people when they need it. | The adolescents linked the item with their life experiences. However, based on the linkages they made, examples of good behavior were added in the brackets, which made the item more understandable and relatable.  A 16-year-old girl said that having good behavior means loving younger ones, never stealing from anyone, never lying to anyone as well as having good thoughts.  Similarly, the younger adolescent aged 13 years said I would choose a lot because I respect and love seniors and they also do the same to me. When my parents went out for some work, the people of my community showed love and called me to their home to have breakfast and behaved with me well. |
| CYRM-R14 | I have opportunities to show others that I am becoming an adult and can act responsibly | तिमीले आफ्नो उमेर अनुसारको काम आफै गरेर अरुलाई देखाउने मौका कत्तिको पाएको छौ ? (उमेरअनुसारको काम जस्तै खाना पकाउने, भाडा माझ्ने, कोठा सफा गर्ने, आफु सुत्ने bed मिलाउने आदि) | How much have you gotten the opportunity to prove yourself to others by doing tasks according to your age by yourself? (For example, cook food, wash dishes, clean house, make your bed) | The groups find difficulty in understanding the items, particularly the terms “adult/mature *(paripakwa)*” and behave responsibly (*jimmewaarpurna vyabahaar)* and suggested replacing them with “according to age (*umer sangai badhdai gayeko*)" and “doing tasks by self (*afno kaam aafai garne*)" respectively. After these modifications, the groups found the item understandable.  The groups said that growing up with age means washing clothes, cooking food, or doing work by ourselves. Likewise, they also said we should do our work by ourselves as our age grows such as taking care of ourselves as well as helping mothers in the kitchen. As we grow up, we get the chance to learn many skills and as a result, we can show our work to other people too. | The adolescents linked this item with their personal experiences, however, with addition of examples of tasks in brackets made the item to comprehend more easily.  One of the younger adolescents aged 11 years said, we have to work based on our age and capacity. I didn’t get enough chance to do work. Some of the work that should be done by our age group includes cleaning the rooms, making my bed, cleaning dishes, and fetching the materials.  Similarly, another adolescent aged 14 years said I live with my maternal uncle and I get a chance to help my uncle in the kitchen for cooking food. Likewise, I do work depending on my capacity such as cleaning the surroundings of the temple and carrying small stones from one place to another, etc.  Likewise, another 15-year-old girl said that even though I am not perfect at cooking food, my *mummy* gave me a chance to cook food. Sometimes, I cook food when my *mummy* gets her period. |
| CYRM-R15 | I feel safe when I am with my family/caregiver(s) | तिमी आफ्नो परिवारसँग हुँदा कत्तिको सुरक्षित महसुस गर्छौ ? (सुरक्षित भन्नाले परिवारसँग हुदा कसैसँग पनि डर नलाग्नु, अरुसंग सजिलै घुलमिल हुन सक्नु, कसैले कुटपिट गर्न नसक्नु आदि) | How safe do you feel when you are with your family? (Feeling safe as in never feeling scared when you are with your family, being able to mingle around with others, others not being able to beat you, etc) | All groups understood this item. They said the question is asking whether we feel safe or not when we are with family members.  They said they don’t feel safe when they go outside alone. When they are with their family no one can beat them or look at them with bad intentions. Similarly, one of the adolescents in the group said when I am not with my *mummy or daddy*, I could be at risk of harassment from adults who smoke and drink. So, if I am with one of the members of the family then I feel safe. | All adolescents related the item to their life experiences.  A 13-year-old adolescent said  when I am with my parents nobody can beat me or threaten me. I feel safe with my parents when I go outside or meet with new people.  Likewise, the older adolescent aged 16 years said, that when I am with my parents, I feel very safe because nobody can touch or hit me. I feel like no one can come to kidnap and touch me when I am with my parents. So, some members stay with me all the time. They don’t leave me alone. However, as one of the adolescents found it difficult to understand the word “*surakshit"* examples of being safe were added in brackets for the clear understanding of the respondents. |
| CYRM-R16 | I have opportunities to develop skills that will be useful later in life (like job skills and skills to care for others) | तिमीलाई भविष्यमा काम लाग्ने सीपहरु तिमीले सिक्ने अवसर पाएको छु जस्तो कत्तिको लाग्छ ? (सिप जस्तै खाना पकाउने, अरुलाई सहयोग गर्ने, लुगा सिलाउने, mobile पसलमा काम गर्ने आदि) | How much do you feel like you have had the opportunity to  learn the skills that will be useful in the future? (Skills such as  cooking food, helping others, stitching clothes, working in mobile  phone store, etc) | All groups understood this item.  The younger adolescents in the groups said skills meant dancing, cooking food, or drawing pictures. If we learn how to cook food then we will get a chance to work in a restaurant in the future. They suggested adding examples like knitting, cooking, and farming under skills.  The older adolescents said, we all have at least one skill some may have cooking skills, some have drawing, and some have mobile repairing skills. We have to show our skills when we get a chance. | All adolescents related the items to their daily lives.  A 14-year-old adolescent said I got a lot of chances to learn everything from my family, not just to study but I got chance to learn skills like cutting glasses, making *tapari* (traditional Nepali leaf plate), collecting firewood, planting rice, cooking food, and sewing.  Another adolescent aged 11 years said in our student life the main duty is to get an education. Besides, that, also has the skill of cooking rice. Additionally, I got a chance to learn agricultural-related works too. Mostly, I cook rice when my mother is out of the home. |
| CYRM-R17 | I enjoy my family’s/caregiver’s cultural and family traditions | आफ्नो परिवारले मनाउने चाडपर्व र परम्परामा तिमि कत्तिको रमाउने गर्छौ ? | How much do you enjoy the festivals and traditions celebrated by your family? | All groups suggested replacing the words “cultural traditions” with “festivals and traditions”. After this replacement of words, all groups understood this item.  The groups related the item and said it is asking whether we enjoy our festivals like *Dashain or Tihar* with our family. In *Dashain* we enjoy putting *tika* on the foreheads and eating delicious food.  One of the older adolescents in the group said some people can’t enjoy the festivals if they have some accidents in the family. I not only enjoy the festivals that my family celebrates. I also celebrate Christmas by going to the church as well as enjoy the Eid festival by going to the mosque. | All adolescents linked the item with their personal life experiences.  An adolescent aged 12 years said most of the time I participate and enjoy the festivals. Especially I enjoy at *Dashain/ Tihar* festival. |

**Table S4.** Item-wise adaptations for RRM

| Item # RRM | Original RRM Item wording | Nepali version | Nepali English Version | Findings from FGDs | Findings from CIs |
| --- | --- | --- | --- | --- | --- |
| RRM1 | I believe in myself | तिमी आफूमा कत्तिको विश्वास गर्छौ ? | How much do you believe in yourself? | The groups understood this item.  The groups said that this question intended to ask, how much confidence we have if we can do something, how much we believe in ourselves to do something, and how much we believe in ourselves to cope and solve problems in difficult situations.  A younger boy in the group said the day before yesterday, I wasn’t understanding math problems but I kept trying. I tried till late at night and I was confident I could solve the problem. Finally, I solved the problem and I felt happy. | The adolescents related the item to their personal life experiences.  A 12-year-old girl said that I don’t believe too much in myself. My mood changes now and then. I have too much belief in my *mom* followed by my family and my friends. I don’t believe in myself because my mood changes now and then. For example, if I said I would go somewhere, in the next few minutes I would say I would not go, my mood keeps changing now and then. That’s why my mom doesn’t ask me any questions like where I am going. If I eat something? What dress I wear?  A 14-year-old boy said I believe that if we try anything, we can achieve it to some extent. I believe I can do it. I believe If I don’t get good results in the academic exam, then I will try next time and the result will be improved. This is the thing I believe in. |
| RRM2 | I can adapt to challenging situations | तिमी कुनैपनि गाह्रो अवस्थामा कत्तिको adjust हुन सक्छौ ? (जस्तै आफु नया ठाँउ वा स्कुलमा जाँदा सजिलै घुलमिल हुन सक्ने, साथिहरु बिच झगडा हुँदा पनि उनीहरुसंग मिलेर बस्न सक्ने, आदि) | How much can you adjust in difficult situations? (For example, being able to mingle around when you go to new place or school, being able to reconcile with friends even after fighting with them, etc) | The adolescents in the first group found it difficult to understand the Nepali word “*dhalnu”* and suggested replacing the Nepali word “*dhalnu*” with another alternative English word “adjust”. They further suggested replacing the Nepali word “*chunautipurna*” with “*garho*” to make it more comprehensive. They also suggested adding examples related to adjusting to challenging situations to make it more comprehensive. The examples added to the question were  “Being able to mingle around when you go to a new place or school, being able to stay together with friends even when you have fights with them, etc.  With these changes in the items and additions, all groups understood the item.  The groups said this question intent to ask how easy and comfortable we can make ourselves in difficult situations. For example, if we move to a new school, we feel difficult and it is also difficult to make new friends. Even in such difficult situations we adjust and cooperate. | All adolescents related the item to their personal life experiences.  A 12-year-old girl said although I was unable to do (any tasks), I kept trying. The day before yesterday, I was suffering from 102°F fever. Despite this, I went to school. No matter how ill I was, I hadn’t stopped going to school. I didn’t miss my study either. That’s why my study is good.  Another 14-year-old girl said how can one adjust when it is very uncomfortable? I couldn’t adjust. When I am uncomfortable, how should I adjust? I can’t adjust. Uncomfortable like when boy touches girl around their chest and shoulder area and during sleep the hand gets bent and becomes difficult.  A 12-year-old girl related the difficult situation to a time of sorrow. For example, there was a lockdown due to covid-19 pandemic. Also, the people’s socioeconomic status varied. During this condition, we didn’t get the things we needed and we couldn’t buy them due to insufficient money. That was a sorrowful situation.  A 14-year-old boy said when someone tells me to move to new places, I feel that I never need to move away from my home and school. I always wish I didn’t need to relocate from my home and school. |
| RRM3 | I find solutions to problems I encounter | तिमीलाई कुनै समस्या आईपर्दा त्यसको समाधान खोज्न कत्तिको सक्छौ ? | How much are you able to look for solutions when you are faced with a problem? | All groups understood this item.  The groups said if we are poor at studying, we can learn at school, and join tuition classes to improve our studies. Likewise, we should be happy with what we have. In the meantime, if we encounter any problems, we don’t need to worry, we should try to find solutions to the problems. | All adolescents related the item to their personal life experiences.  A 12-year-old adolescent said that within 10 minutes, 15 minutes, or 30 minutes, all ideas come to me to solve the problems. If any problems arise, I could solve them. When I fight with friends, I convince them. Even when friends weren’t convinced, and initially I hadn’t idea how to convince them, in that situation, I generated ideas quickly to convince them. Similarly, for the project work assigned by the teachers, I quickly generated ideas to do the project work. And some school tasks need self-exploration, and self-imagination to complete. Once our teacher assigned the same task to self-explore, self-imagine, and write and I completed it. Similarly, in grade five I made a drawing by self-imagination.  A girl aged 14 years said that if I faced any problems I could seek support from my mom, dad, stepdad, or other family members. I can speak with any family member about the problems I face. When I had my first menstrual period and during this phase, there was a lockdown (due to covid-19 pandemic) and I was staying with my grandmother. As that was my first menstrual period, I felt very uncomfortable. I sought advice from my mom by calling her over the phone. I care for myself accordingly. Similarly, sometimes we found difficult to study and, in that situation, we can seek support from our teachers or our elder sisters at home. |
| RRM4 | I can keep going despite difficulties | तिमीलाई गाह्रो भएता पनि हार नमानी अघि बढ्न कत्तिको सक्छौ ? | How much are you able to proceed forward and not give up even when you are experiencing difficulty? | All groups understood the item. However, the first group suggested replacing the words "*kathinaaiihru bhaeko awastha*" (fora difficulties) with "*gaarho*" and adding an extra word "*haar namaani*” (without giving up). These slight changes made the item more comprehensible to the groups.  The groups said that the item is asking about even in a difficult situation, how much one can keep moving forward. For example, even if one fails in the exam, she/he should keep studying. If she/he can’t study in this school, she/he can study in another school. | All adolescents related the item to their personal life experiences.  A 14-year-old boy said that although some tasks are difficult, we need to do it. No matter how much difficult the task is in the present, if it is useful for the coming days then we need to complete the task. For example, as a student, if I feel math subject is difficult to understand then we need to invest more time to study math. For the first attempt, although I didn’t understand a lot, I am investing time in it. If I keep trying and studying hard then at least I might pass the math subject.  A girl aged 12 years said I can move forward; I can do a lot. I am the person who wins even after facing the losses. And I never lose. Even when I lose, I keep going. I don’t feel worried. Sometimes I feel worried during exam time. What to do and how to do things during those worrying times depend upon the situation. |
| RRM5 | I can cope with competing demands (for my time or attention) | तिमीले सधैजसो गर्ने भन्दा धेरै कामहरु गर्नु पर्दा ती कामहरु सम्हाल्न कत्तिको सक्छौ ? (जस्तै tuition पढ्ने, खेल्ने, homework, खाना पकाउने आदि) | How well are you able to deal with having more tasks than usual? (Tasks such as tuition classes, playing, homework, cooking food, etc) | All adolescents understood the item. The adolescent understood this item. However, they suggested adding examples of competing demands in brackets to make the item more comprehensive such as tuition, playing, homework, cooking food, etc.  The groups said this question intended to ask sometimes the amount of work may be higher than we usually did, and sometimes we need to complete many tasks at the same time. In that situation how much, we can balance ourselves and manage our time to complete the tasks?  An adolescent boy in the group said if I am working on some tasks at home and I need to do other tasks at the same time for example while completing homework, others task such as looking for younger siblings may need to do. In such a situation I need to manage some time to complete household work (looking for younger siblings). | All adolescents related the item to their personal life experiences.  A girl aged 14 years said I can’t handle many tasks. I need to go tuition classes; I have a lot homework to complete. Even I kept doing homework all time, I can’t complete it.  A 14-year-old boy said that where I stay (home) there are a lot of friends, it is enjoyable to work together with friends. I think even when tasks are difficult, we can complete it quickly working together. That’s why I think even when there are multiple tasks or more tasks, it can be simpler, enjoyable, and doable to work together in a team. |
| RRM6 | Even when there are setbacks or obstacles, I am hopeful about my future | जिवनमा गाह्रो/अप्ठ्यारोहरु आएतापनि तिमी आफ्नो भविष्यप्रति कत्तिको आशा गर्छौ ? | How much are you hopeful about your future even when there are difficulties in your life? | All groups understood the item after the recommended replacement of the words was made. *Awarodh, badhaadchan* (setbacks or obstacles) was replaced with ”*jiwanama garho/apthayaroharu*” (difficulties in life.  The groups said this question intends to ask, how much we can keep our hope alive for the future when we encounter some common difficulties in our lives. For example, if someone wishes to travel to foreign countries but their parents don’t have money to allow him/her to visit foreign countries. In such a situation, he/she should plan to earn money by himself/herself and achieve his/her aim to travel to foreign countries. He/she should be hopeful for such things. | All adolescents related the item to their personal life experiences.  A 14-year-old girl said I am very much hopeful during difficult times. When I see my mom and dad working hard and their struggle, I feel motivated to do good work in the future and I am hopeful everything will go well. |
| RRM7 | I am generally in control of my emotions | तिमी प्रायजसो आफ्नो feelings लाई कत्तिको सम्हाल्न (control गर्न ) सक्छौ? (जस्तै खुःसी, दुःखी, निरास, डाहा, आदि भएको बेला आफुलाई सम्हाल्नु) | How much are you able to control your feelings? (For example being able to control yourself when you are feeling happy, sad, upset, jealous, etc ) | The adolescents in the groups found it difficult to understand the Nepali term “*bhaawanaa”* and suggested changing it to “feelings” which is more common and easy to understand. They also suggested adding examples of feelings such as happy, sad, upset, jealous, etc. to make the item more comprehensible.  The groups understood the item after such change of word and addition of examples of feelings, and said that the item is asking most of the time, how much you were able to control yourself when you were happy or sad. For example, if you secure the first position in the academic exam then you may be extremely happy and you may stop concentrating on study. Then you may fail next year. | All adolescents related the item to their personal life experiences.  A 14-year-old girl said I can control my feelings a little bit. When I feel something, I share it with my friends. I think when I feel happy or hurt, I should speak about it.  A boy aged 13 years said that I can control my feelings a lot. For example, when I am happy, I feel joy. When I receive new toys like remote-controlled vehicle toys and cars, I feel joyous. And I show these toys to my friends. When I felt sad, I felt sad internally. I think, when I am sad, my friends would ask me why I am sad. That’s why I hide my sadness and limit it internally. |
| RRM8 | I take pride in things I have achieved | तिमीले सोचेको काम पुरा गरेकोमा कत्तिको गर्व गर्छौ (जस्तै पढाईमा success हुँदा, खेलहरु जित्न, नयाँ साथीहरु बनाउने, स्वादिष्ट परिकार पकाउने आदी) ? | How proud do you feel of yourself when you are able to accomplish the things that you wanted to? (For example, when you are successful in your studies, when you win in sports, when you are able to make new friends, when you cook a delicious meal, etc) | All groups understood this item. However, they suggested adding examples of accomplishments to make the item more comprehensive. Examples include when you are successful in your studies, when you win in sports, when you can make new friends, when you cook delicious meals, etc.  The groups said that if you make something good like making delicious food, or winning a trophy then in our home our parents support and motivate us to do even better and during those situations, it feels proud. Likewise, if we find it difficult to solve any math problem, but if we keep trying to solve this problem and finally solve it. Then also we feel proud. | All adolescents related the item to their personal life experiences.  A 12-year-old girl said when I accomplished the task I had thought of, I felt happy, I felt a bit proud of myself. When I succeed in my studies, I feel proud. When I achieved whatever, I had thought of, I felt extremely happy. I consider myself above all. |
| RRM9 | When faced with difficulties, I rise to the challenge | तिमीलाई गाह्रो हँदा त्यस्तो अवस्थासँग सामना गर्न कत्तिको खोज्छौ ? | When you have difficulty, how much do you try to face those situations? | The groups found Nepali terms “*kathinaaiharu*” and “*chunautiharu*” a bit difficult to understand and suggested replacing them with  “*garhoo awastha”* (difficult situations) *saamanaa garna kattiko khojchhau*”. They suggested replacing the phrase “how much you are interested to take the challenges” to “how much you try to face such situation”.  All groups understood the item after these slight changes.  They said that this question intended to ask how much we want to face the difficult situation. When difficult situations arise, whether we stay just as before or try to solve the difficult situations. For example, if we have to face any challenges like sports or study-related challenges. In sports, if we are losing then we need to accept ourselves and try hard to win next time. | All adolescents related the item to their personal life experiences.  A boy aged 13 years said I try to solve my problems a lot. This is because I try to solve my problems myself. For example, when I don’t have money to buy copy, I borrow money from my friends. And I return the money to my friends the next day or the day after next day. In this way I solve my problems. |
| RRM10 | I can find meaning in my life | तिमी आफ्नो जीवनको उद्देश्य कत्तिको बुझ्न सक्छौ ? | How much are you able to understand the purpose of your life? | All groups understood the item.  The groups related the item by narrating a few examples.  In our class, there is a boy named Dinesh (name changed). He is perfect in drawing. If he sets his goal to be one of the best artists and understands his aim to be an artist then he can do something in his life and can earn his living.  Similarly, they said that this item is asking if you can understand what you will be in the future (can you understand what goal you set for yourself for your future)? | All adolescents related the item to their personal life experiences.  A 14-year-old girl said I can understand the purpose of my life a little. Till now, I don’t have any aim for my future that’s why I can understand a little only. The purpose of life is life’s future goal or aim. For example, I want to be a social worker in the future. I can understand the purpose of my life a little because I haven’t planned much for the future. |

**Table S5.** Factor Loadings of the Three Factor Solution of CYRM-R Nepali Items for PAF with Oblimin Rotation

| Tool/Item | Factor 1 | Factor 2 | Factor 3 | Communalities |
| --- | --- | --- | --- | --- |
| CYRM-R1 | 0.22 | 0.10 | **0.31** | 0.28 |
| CYRM-R2 | **0.41** | -0.16 | 0.12 | 0.17 |
| CYRM-R3 | 0.12 | 0.29 | 0.19 | 0.23 |
| CYRM-R4 | **0.75** | 0.09 | 0.02 | 0.66 |
| CYRM-R5 | **0.33** | -0.03 | **0.41** | 0.40 |
| CYRM-R6 | 0.29 | 0.03 | **0.32** | 0.30 |
| CYRM-R7 | 0.20 | 0.00 | **0.45** | 0.33 |
| CYRM-R8 | 0.20 | 0.02 | **0.36** | 0.26 |
| CYRM-R9 | -0.11 | 0.03 | **0.77** | 0.53 |
| CYRM-R10 | 0.02 | 0.23 | **0.60** | 0.55 |
| CYRM-R11 | **0.50** | 0.00 | 0.23 | 0.43 |
| CYRM-R12 | 0.12 | -0.09 | **0.65** | 0.47 |
| CYRM-R13 | 0.00 | 0.02 | **0.59** | 0.36 |
| CYRM-R14 | -0.07 | **0.68** | 0.12 | 0.49 |
| CYRM-R15 | **0.86** | 0.03 | -0.07 | 0.71 |
| CYRM-R16 | 0.07 | **0.89** | -0.04 | 0.83 |
| CYRM-R17 | **0.46** | 0.10 | 0.15 | 0.37 |

Note: Factor correlations between Factor 1 and 2 (0.52), Factor 1 and 3 (0.52), and Factor 2 and 3 (0.41)

**Table S6.** Factor Loadings of the Four Factor Solution of CYRM-R Nepali Items for PAF with Oblimin Rotation

| Tool/Item | Factor 1 | Factor 2 | Factor 3 | Factor 4 | Communalities |
| --- | --- | --- | --- | --- | --- |
| CYRM-R1 | 0.25 | 0.21 | **0.37** | -0.01 | 0.36 |
| CYRM-R2 | -0.02 | -0.09 | **0.84** | 0.06 | 0.72 |
| CYRM-R3 | 0.16 | **0.33** | 0.09 | 0.05 | 0.24 |
| CYRM-R4 | 0.05 | 0.15 | 0.11 | **0.65** | 0.64 |
| CYRM-R5 | **0.42** | -0.02 | -0.04 | **0.37** | 0.43 |
| CYRM-R6 | **0.33** | 0.08 | 0.02 | 0.26 | 0.31 |
| CYRM-R7 | **0.41** | 0.06 | 0.16 | 0.11 | 0.33 |
| CYRM-R8 | **0.37** | 0.01 | -0.05 | 0.26 | 0.28 |
| CYRM-R9 | **0.73** | 0.03 | 0.01 | -0.07 | 0.52 |
| CYRM-R10 | **0.55** | **0.30** | 0.22 | -0.09 | 0.58 |
| CYRM-R11 | 0.26 | 0.01 | -0.03 | **0.53** | 0.46 |
| CYRM-R12 | **0.68** | -0.11 | -0.10 | 0.20 | 0.53 |
| CYRM-R13 | **0.56** | 0.07 | 0.09 | -0.05 | 0.36 |
| CYRM-R14 | 0.10 | **0.67** | -0.08 | -0.01 | 0.48 |
| CYRM-R15 | -0.05 | 0.06 | 0.06 | **0.84** | 0.76 |
| CYRM-R16 | -0.05 | **0.87** | -0.03 | 0.10 | 0.80 |
| CYRM-R17 | 0.12 | 0.19 | 0.25 | **0.30** | 0.39 |

Note: All four factors show moderate positive correlations (r =0.19–0.48).

**Table S7.** Comparison of 1- to 4-Factor EFA Models for Nepali CYRM-R

| Metric/Model | 1-Factor Model | 2-Factor Model | 3-Factor Model | 4-Factor Model |
| --- | --- | --- | --- | --- |
| Variance | 33% | 38% | 43% | 48% |
| RMSR | 0.08 | 0.07 | 0.05 | 0.04 |
| Off-diagonal fit | 0.94 | 0.96 | 0.97 | 0.98 |
| Factor score correlation | 0.95 | 0.91-0.93 | 0.91-0.93 | 0.87-0.93 |
| Minimum factor score correlation | 0.80 | 0.67-0.75 | 0.67-0.74 | 0.53-0.72 |
| Item loadings | 0.35-0.71 | 0.28-0.84 | 0.12-0.89 | 0.05-0.87 |
| Cross-loadings | None | Some moderate cross-loadings (0.36-0.57) | Some low and cross-loadings (0.12-0.45) | More cross-loadings, some items load weakly (<0.02) |
| Factor interpretability | Single construct | Two interpretable factors; most items load clearly | Three factors; one factor has few items, some cross-loadings | Four factors; some factors are unstable (a few items, complex) |

Note: Acceptable fit thresholds: Chi-Square Minimum/degree of freedom (χ²/df) ≤ 3, Goodness of Fit Index (GFI)/Comparative Fit Index (CFI)/Normative Fit Index (NFI) ≥ 0.90, Root Mean Square Error of Approximation (RMSEA) ≤ 0.08
